# Final Selection of Quality Protein Popcorn Hybrids

**DOI:** 10.3389/fpls.2021.658456

**Published:** 2021-03-24

**Authors:** Leandra Parsons, Ying Ren, Abou Yobi, Ruthie Angelovici, Oscar Rodriguez, David R. Holding

**Affiliations:** ^1^Department of Agronomy and Horticulture, University of Nebraska–Lincoln, Lincoln, NE, United States; ^2^Center for Plant Science Innovation – Beadle Center for Biotechnology, University of Nebraska–Lincoln, Lincoln, NE, United States; ^3^Division of Biological Sciences and Interdisciplinary Plant Group, University of Missouri, Columbia, MO, United States; ^4^ConAgra Brands, Springfield, IN, United States

**Keywords:** hybrid-analysis, index-selection, maize-breeding, popcorn, *opaque-2*

## Abstract

Quality Protein Popcorn (QPP) BC_2_F_5_ inbred lines were produced through an interpopulation breeding system between Quality Protein Maize dent (QPM) and elite popcorn germplasm. In 2019, five QPP F_1_ hybrids were selected for further evaluation due to superior agronomics, endosperm protein quality, and popping quality traits. Though these BC_2_F_5_ QPP hybrids were phenotypically similar to their popcorn parents, the QPP cultivars conveyed slightly inferior popping characteristics when compared to the original popcorn germplasm. The objective of this study was twofold. First, BC_2_F_5_ inbred lines were crossed to their popcorn parents and BC_3_F_4_ inbred lines were produced for hybridization to test the agronomic, protein, and popping trait effects from an additional QPP by popcorn backcross. Second, BC_2_- and BC_3_-hybrids were simultaneously evaluated alongside ConAgra Brands^®^ elite cultivars and ranked for potential commercialization in the spring of 2020. These 10 QPP hybrids were grown alongside five ConAgra Brands^®^ elite popcorn cultivars in three locations and agronomic, protein quality, and popping quality traits were evaluated. Significant improvements in popcorn quality traits were observed in the QPP BC_3_ cultivars compared to their BC_2_ counterparts, and yield averages were significantly lower in BC_3_-derived QPP hybrids compared to the BC_2_ population. Protein quality traits were not significantly different between QPP backcrossing populations and significantly superior to ConAgra elite popcorn varieties. Utilizing a previously published ranking system, six QPP hybrids, three from the BC_2_F_5_ population and three from the BC_3_F_4_ population, were evaluated as candidates for final selection. The successful evaluation and ranking system methodology employed is transferable to other hybrid production and testing programs. Incorporating this analysis with concurrent sensory studies, two QPP hybrids were chosen as premier cultivars for potential commercialization.

## Introduction

Popcorn [*Zea mays* L. ssp. *everta* (Sturt.) Zhuk] is a type of flint corn characterized by its ability to expand and form light flakes under high heat. Popcorn has been enjoyed as a direct-to-consumer product in the United States for more than a century, and in 2013, the popcorn industry revitalized from a two-decade retailing plateau owing to growing consumer demand for a healthier, innovative snack food option and increased diversity in the popcorn market (Smith et al., 2004; Topping, 2011; Mordor Intelligence, 2018). Intra-subspecies crosses between dent maize and popcorn, one avenue for increasing diversity in the popcorn germplasm pool, have shown to enhance popcorn’s agronomic fitness and flavor profile at the cost of deficient popcorn quality traits such as popability and expansion volume (Robbins and Ashman, 1984; Sprague and Dudley, 1988; Dofing et al., 1991; Ziegler and Ashman, 1994). To negate these undesired side effects, a study in 2018 described an inter-subspecies breeding program crossing highly vitreous dent Quality Protein Maize dent (QPM) varieties with proprietary popcorn lines to produce highly vitreous, high lysine Quality Protein Popcorn (QPP) BC_2_F_5_ inbred lines (Ren et al., 2018). Concurrent to rapidly restoring popcorn traits, these unique popcorn inbred lines carried the *opaque-2* homozygous recessive mutation and conferred a 1.5-2 fold increase in kernel endosperm lysine levels compared to the original popcorn parents (Ren et al., 2018). This proof-of-concept study supported the positive correlation between kernel endosperm vitreousness, the hard and translucent endosperm phenotype, and popcorn quality traits, a previously published but majorly unexplored concept (Hoseney et al., 1983; Matz, 1984; da Silva et al., 1993; Smith et al., 2004; Babu et al., 2006). Methods involved in this study included a phenotypic assessment of vitreousness, genotypic marker-assisted-selection for the *opaque-2* allele, and proteomic evaluation through endosperm protein extraction and SDS-PAGE (Ren et al., 2018). Though modifier genes conferring vitreousness in *opaque-2* carrying lines still remain largely unknown, a 2016 study confirmed that the over-expression of the 27-kd γ-zein maize endosperm storage protein, a known requirement for restoring vitreousness in *opaque-2* carrying lines, was due to a genetic duplication of the 27-kd γ-gene (Liu et al., 2016). Since the rest of the endosperm modifier genes are unspecified (though genetic locations have been postulated), phenotypic evaluation of vitreousness and zein profiling still serve as the best means for selecting vitreousness, and consequently popping traits, in an *opaque-2* background (Holding et al., 2008; Wu et al., 2010; Holding, 2014; Parsons et al., 2020).

To further develop this proof-of-concept intraspecies breeding program, 12 BC_2_F_5_ QPP inbreds were hybridized in the summer of 2018 to produce 132 QPP F_1_ lines. After initial observation, 44 QPP hybrids were selected for further pre-screening analysis of agronomic, protein, and popcorn quality traits. In the summer of 2019, these 44 hybrids were grown in multiple locations and 14 traits were evaluated for selection of five superior BC_2_F_5_ QPP hybrids (Parsons et al., 2020). Quantitative positive correlations between popcorn expansion volume, popability, and endosperm vitreousness were measured, and the results further emphasized the preliminary requirement for highly vitreous dent parents in a successful popcorn by dent maize subspecies crosses (Parsons et al., 2020). The five selected QPP BC_2_F_5_-derived hybrids had relatively superior agronomics, elevated lysine levels in the kernels, and the most optimal popcorn quality traits compared to the rest of the assessed hybrids.

Though the QPP hybrids were phenotypically indistinct from the original popcorn lines and demonstrated comparatively superior agronomics and adequate popping characteristics, previous studies have suggested that multiple rounds of backcrossing aid in restoring popping expansion volume (Babu et al., 2006). The five elite BC_2_F_5_ QPP hybrids chosen in 2019 had insignificant differences in popability (number of unpopped kernels/number of kernels tested), but slightly lower popping expansion volume compared to original popcorn germplasm (Parsons et al., 2020). To test the potential improvement of QPP popcorn quality traits, specifically popping expansion volume, by backcrossing, BC_2_F_5_ QPP inbreds were again backcrossed to elite popcorn parental lines, and *opaque-2* carrying, phenotypically vitreous BC_3_F_4_ QPP lines were produced in the fall of 2019. These BC_3_F_4_ inbreds were selectively crossed to produce the five pre-selected QPP hybrids from the 2019 analysis of BC_2_F_5_ crosses. In the summer of 2020, these 10 QPP hybrids, five BC_2_F_5_ and five BC_3_F_4_ derived, and five selected ConAgra Brands^®^ popcorn hybrid cultivars were grown to compare QPP and ConAgra Brands^®^ popcorn cultivars based on agronomic, popcorn quality, and protein quality traits. Overall, a trade-off between popcorn quality and agronomic traits was observed. Protein quality traits were not significantly different between QPP backcrossing populations and significantly superior to ConAgra elite popcorn varieties. This study utilized a previously published ranking system concurrently with experimental data to select lines for large-scale field trials. The detailed, successful utilization of this ranking system in this study emphasizes its conveyable utility for other hybrid testing programs in evaluation and selection of premier hybrids fit for commercialization. After ranking, six QPP hybrids were selected as forerunner candidates for final selection. Overall, incorporating this selection with sensory analysis, two best QPP hybrids were chosen as premier candidates for potential commercialization.

## Materials and Methods

### Plant Materials

#### BC_2_F_5_ Inbred QPP Lines

BC_2_F_5_ inbred QPP lines were produced by a QPM by popcorn backcross breeding program as described in Ren et al. (2018). Briefly, QPM lines CML154Q, Tx807, and K0326Y were crossed to ConAgra Brands^®^ proprietary popcorn inbred lines labeled P1–P4 (proprietary names withheld) in 2013. Original ConAgra Brands^®^ popcorn inbred lines were provided by ConAgra Brands^®^, K0326Y QPM dent maize was provided by Hans Gevers (Gevers and Lake, 1992), and CML154Q and Tx807 were provided from the North Central Regional Plant Introduction Station (Ren et al., 2018). To produce the BC_2_F_5_ inbred QPP lines, F_1_ hybrids were backcrossed twice to the popcorn parent and self-pollinated five times rendering 0.39% theoretical heterozygosity (Ren et al., 2018).

#### BC_3_F_4_ Inbred QPP Lines

BC_3_ lines were produced by an additional cross of female ConAgra^®^ popcorn lines with male BC_2_F_5_ QPP inbred lines during the summer of 2018. These BC_3_F_1_ lines were self-pollinated in the winter of 2018 and the BC_3_F_2_ seed segregated for the QPM *opaque-2* allele. Homozygous recessive *opaque-2* kernels were selected through SDS-PAGE and marker-assisted selection (as detailed below) and subsequently self-pollinated twice. BC_3_F_4_ seed was produced in the summer of 2019 concurrent with BC_2_F_5_ QPP hybrids analysis. Assuming a theoretical genetic contribution of popcorn to dent maize as 93.75 and 6.25%, respectively, and the homozygosity of an F_4_ at 93.75%, the resultant heterozygosity in the BC_3_F_4_ inbred lines is equivalent to the BC_2_F_5_ lines at 0.39%. Comparatively, an F_8_ inbred line confers the same heterozygosity of 0.39% (Collard et al., 2005; Uptmoor et al., 2006; Gupta et al., 2010).

#### BC_2_F_5_, BC_3_F_4_, and ConAgra^®^ Brands F_1_ Hybrid Seed

After the 2019 summer field trials, five QPP BC_2_F_5_ hybrids were selected for further testing: Hybrid 20 (QPP BC_2_F_5_ Inbred 6 x QPP BC_2_F_5_ Inbred 10), Hybrid 25 (QPP BC_2_F_5_ Inbred 9 x QPP BC_2_F_5_ Inbred 3), Hybrid 28 (QPP BC_2_F_5_ Inbred 9 x QPP BC_2_F_5_ Inbred 6), Hybrid 38 (QPP BC_2_F_5_ Inbred 10 x QPP BC_2_F_5_ Inbred 5), and Hybrid 43 (QPP BC_2_F_5_ Inbred 10 x QPP BC_2_F_5_ Inbred 11) (Parsons et al., 2020). In the spring of 2020, BC_2_F_5_ and BC_3_F_4_ hybrids of the chosen crosses were produced and F_1_ seed was harvested. These QPP cultivars were grown alongside five ConAgra check hybrids and varietals in the summer of 2020. {Popcorn parent 1 x Popcorn parent 2} seed and its reciprocal seed were produced in the spring of 2020 alongside QPP hybrids, and {Popcorn parent 1 x Popcorn parent 3} seed and two check ConAgra varietals were supplied by ConAgra Brands^®^. In all, 15 cultivars were planted in the summer of 2020 and numerically named 1–15 in order of BC_2_F_5_ hybrids, BC_3_F_4_ hybrids, and ConAgra test cultivars, respectively (Table 1).

**TABLE 1:** Description of cultivars tested in 2020 summer trials.

Cultivar	Previous nomenclature	Ref. No.	Pedigree
BC_2_F_5_ F_1_ Hybrid	Hybrid 20	H1	(PP2 x (K0326Y x PP2))F_5_ x (PP1 x (CML154Q x PP1))F_5_
	Hybrid 25	H2	(PP1 x (CML154Q x PP1))F_5_ x (PP3 x (CML154Q x PP3))F_5_
	Hybrid 28	H3	(PP1 x (CML154Q x PP1))F_5_ x (PP2 x (K0326Y x PP2))F_5_
	Hybrid 38	H4	(PP1 x (CML154Q x PP1))F_5_ x (PP2 x (K0326Y x PP2))F_5_
	Hybrid 43	H5	(PP1 x (CML154Q x PP1))F_5_ x (PP3 x (Tx807 x PP3))F_5_
BC_3_F_4_ F_1_ Hybrid	Hybrid 20	H6	(PP2 x ((PP2 x (K0326Y x PP2))F_5_)F_4_ x (PP1 x ((PP1 x (CML154Q x PP1))F_5_)F_4_
	Hybrid 25	H7	(PP1 x ((PP1 x (CML154Q x PP1))F_5_)F_4_ x (PP3 x ((PP3 x (CML154Q x PP3))F_5_)F_4_
	Hybrid 28	H8	(PP1 x ((PP1 x (CML154Q x PP1))F_5_)F_4_ x (PP2 x ((PP2 x (K0326Y x PP2))F_5_)F_4_
	Hybrid 38	H9	(PP1 x ((PP1 x (CML154Q x PP1))F_5_)F_4_ x (PP2 x ((PP2 x (K0326Y x PP2))F_5_)F_4_
	Hybrid 43	H10	(PP1 x ((PP1 x (CML154Q x PP1))F_5_)F_4_ x (PP3 x ((PP3 x (Tx807 x PP3))F_5_)F_4_
ConAgra^®^ Brands Popcorn	PP1 x PP2	H11	PP1 x PP2
	PP2 x PP1	H12	PP2 x PP1
	PP1 x PP3	H13	PP1 x PP3
	Cultivar 1	H14	Commercial Line (CL) 1
	Cultivar 2	H15	Commercial Line (CL) 2

*15 total cultivars were grown and evaluated in the summer of 2020. “Previous Nomenclature” as described in Parsons et al. (2020) is in reference to QPP pedigree. For simplicity, reference numbers H1–H15 were utilized for identification of hybrids in this analysis.*

### 2020 Field Design

The 15 selected cultivars were grown in three locations over the summer of 2020. Seed was sown on April 30 in Lincoln, Nebraska (40°50′11.6”N 96°39′42.4”W DMS), May 1 in Mead, Nebraska (41°08′51.6”N 96°27′04.7”W DMS), and May 5 in Colby, Kansas (39°22′50.7”N 101°03′33.0”W DMS) in collaboration with Kansas State University’s Northwest Research-Extension Center. Trials were designed in a Generalized Randomized Block Design (GRBD) with three experimental unit (EU) replications per location. EUs were 17 feet (5.18 m) by four row (10 feet or 3 m) plots planted at ∼34,500 plants/acre (8.53 plants/m^2^) and each plot was separated on all sides by six to eight rows of dent border corn to prevent popcorn cross-pollination (popcorn seed utilized was cross-incompatible with bordering dent maize pollen). The center two rows of EUs were machine harvested and random ears from the fourth row were hand-harvested for analysis.

### Zein and Non-Zein Protein Extraction and SDS-PAGE Profiling

F_1_ hybrid seeds from the 2020 field trials for all experimental crosses were subjected to zein and non-zein protein analysis as previously described (Wallace et al., 1990; Ren et al., 2018; Parsons et al., 2020). QPP F_1_ and F_2_ hybrid seed produced from BC_2_F_5_ and BC_3_F_4_ inbred lines were tested to verify a QPM-patterned proteome of high 27-kD γ-zein and low α-zeins. The specific procedures used for both zein and non-zein analysis are described in Parsons et al. (2020). Briefly, equal amounts of raw kernel flour were introduced to a borate extraction buffer and the protein supernatant was extracted. Zein and non-zein fractions were separated by adding 70% ethanol and incubating overnight. The soluble zein and non-soluble non-zein fractions from identical amounts of flour were separated and proteins were profiled using acrylamide SDS-PAGE (Wallace et al., 1990).

### Validating *o2o2* Genotype in QPP Inbreds

QPP BC_2_F_5_ and BC_3_F_4_ inbred lines utilized for hybrids, Inbreds 3, 5, 6, 9, 10, and 11, were genotyped for *o2o2* validation using *opaque-2* in-gene marker umc1066 and flanking marker bnlg1200 (Babu et al., 2005; Ren et al., 2018; Parsons et al., 2020). Inbreds 3, 9, 10, and 11 were genotyped by bnlg1200, while Inbreds 5 and 6 were genotyped by in-gene marker umc1066. Polymerase chain reaction (PCR) was carried out according to Ren et al. (2018) except TaKaRa Ex Taq DNA polymerase was used. Annealing temperatures for umc1066 varied between 60 and 63°C and held at approximately 55°C for bnlg1200. For DNA, 2-week-old leaf tissue was collected and DNA extracted according to a previously published procedure (Holding et al., 2008). Crude DNA was diluted 20-fold with double distilled or autoclaved water for an average concentration of 50 ng/μL.

### Trait Analysis

Cultivars were harvested with a Kincaid XP-8 two-row plot combine harvester capable of estimating test weight (lbs/bu), plot weight (lbs), and moisture content. Yield estimates were determined by Eqs 1 and 2 and pounds of dry matter per bushel was measured for kernel size comparisons, as shown in Eq. 3.

(1) $$Y\,i\,e\,l\,d\,\left(\frac{p\,o\,u\,n\,d\,s\,o\,f\,d\,r\,y\,m\,a\,t\,t\,e\,r}{f\,e\,e\,t^{2}}\right)=\frac{P\,l\,o\,t\,w\,e\,i\,g\,h\,t\,\left(p\,o\,u\,n\,d\,s\right)*\left(1-m\,o\,i\,s\,t\,u\,r\,e\,p\,e\,r\,c\,e\,n\,t\,a\,g\,e\right)}{85\,f\,e\,e\,t^{2}}$$

(2) Y⁢i⁢e⁢l⁢d⁢(56⁢l⁢b⁢b⁢u⁢s⁢h⁢e⁢l⁢a⁢t⁢ 15.5%⁢m⁢o⁢i⁢s⁢t⁢u⁢r⁢ea⁢c⁢r⁢e)=(P⁢l⁢o⁢t⁢w⁢e⁢i⁢g⁢h⁢t⁢(p⁢o⁢u⁢n⁢d⁢s)*(1-{m⁢o⁢i⁢s⁢t⁢u⁢r⁢e⁢p⁢e⁢r⁢c⁢e⁢n⁢t⁢a⁢g⁢e-.155}*.012)56)*512.5

(3) $$\frac{p\,o\,u\,n\,d\,s\,o\,f\,d\,r\,y\,m\,a\,t\,t\,e\,r}{v\,o\,l\,u\,m\,e\,t\,r\,i\,c\,b\,u\,s\,h\,e\,l}=T\,e\,s\,t\,w\,e\,i\,g\,h\,t\,\left(\frac{p\,o\,u\,n\,d\,s}{v\,o\,l.b\,u\,s\,h\,e\,l}\right)*\left(1-m\,o\,i\,s\,t\,u\,r\,e\,p\,e\,r\,c\,e\,n\,t\,a\,g\,e\right)$$

Equation 1 estimated the amount of dry matter accumulated from each EU, while Eq. 2 evaluated the yield of the plots on a 15.5% grain moisture bushel (the standard moisture value of a dry maize bushel) basis. A value of 1.2% shrinkage due to expected water loss was incorporated into the equation (Hicks and Cloud, 1991). The factor “512.5” denotes the fold increase in square feet required for acre units. Equation 3 aided in estimating kernel size. Test weight was mechanically measured by the plot combine, and an average measurement in pounds was identified and returned. The yield estimate of Eq. 1 was used in the 2020 Ranking System (detailed below). Final values from Eqs 1–3 discussed below were converted into metric units.

Approximately two pound (∼1000 g) sub-samples were obtained from the center two rows of each EU to measure vitreousness, expansion volume, popability, and flake morphology. Kernel vitreousness was assessed on a scale of 1–7 as previously described (Parsons et al., 2020). Five ears were randomly hand-harvested from the fourth row of every EU for one average ear length measurement per EU and amino acid profiling of the endosperm proteome. Three measurements of plant height were recorded and averaged for one height measurement per EU. Roughly 250 g of machine-harvested seed from each EU (135 total samples) were placed in a conditioning room set at 14% moisture for 6 weeks for moisture equilibration prior to popping quality tests. After equilibration, the 250 g samples were popped and measured for expansion volume (cubic centimeters per grams), popability (number of unpopped kernels/total number of kernels subjected to popping mechanism expressed as a percentage), and flake size index (FSI) estimates. FSI was estimated using Eq. 4:

(4) $$OCFSI=\left(OE^{*}250\right)/\left(\left(\left(\frac{K\,C}{10}\right)*Charge\right)-UPK\right))$$

The Oil Crude FSI (OCFSI) is an estimate of an average individual kernel’s flake expansion under oil-popping conditions. “*OE*” is the expansion volume measured in a graduated cylinder (0–50 mL) of expansion volume per gram in cubic centimeters. The “*KC*” value is the number of kernels in a random sample of 10 g (the counted samples’ weight can be modified and the denominator changed accordingly). *Charge* is the sample weight charged into the oil popper (250 g), and *UPK* represents the total number of unpopped kernels in the 250 g sample. By multiplying total expansion (OE) by the sample weight, and dividing the product by an estimated popped kernel number, the FSI served as an individual measure of popped kernel expansion. Measurement of sample expansion volume, popability, and OCFSI estimates were accomplished utilizing ConAgra Brands test oil popper (Cretors 120V, 60 Hz, 2300 Watts, Cretors & Co, Chicago, IL, United States), and facilities in Brookston, Indiana. Categorical observation of flake morphology as either mushroom, butterfly, or mixed was ascertained.

Free and protein-bound amino acid profiles of all tested cultivars were analyzed at the University of Missouri according to previously published procedures (Angelovici et al., 2013; Yobi and Angelovici, 2018). Six samples from each cultivar, three in raw kernel powder and three in air-popped flake forms, were analyzed. Raw flour and air popped flake samples were prepared according to previously described procedures (Parsons et al., 2020). Briefly, for popped samples, air-popped flakes were frozen in liquid nitrogen and then grounded using a mortar and pestle until fine powder. Free and protein-bound amino acids from B73 kernel flour were also measured for reference.

### Statistical Analysis

Cultivar trait estimates were analyzed by the statistical model given by Eq. 5:

(5) $$y_{i\,j\,k}=\mu +\beta_{i}+\tau_{j}+\left(\beta \,\tau_{i\,j}\right)+\varepsilon_{i\,j\,k}$$

In which *y*_*ijk*_ is the cultivar’s response, μ is the overall mean, β_*i*_ is the block, or locational, effect, τ_*j*_ is the treatment, or cultivar, effect, (βτ_*ij*_) is the location^∗^treatment interaction, and ε_*ijk*_ is the experimental error (Addelman, 1969). Type II sums of squares was used to compute the analysis of variance, and the treatment effect was fixed. The Central Limit Theorem was assumed for normality of the data. R Software was used to conduct all analysis including trait correlations and Tukey’s honest significant differences (R Core Team, 2018). The R package “ggfortify” was used for PCA analysis of all protein-bound amino acid values through singular value decomposition and the “prcomp” function (Tang et al., 2016).

### Cultivar Index Selection: 2020 Ranking System

As shown in Eq. 6, the 2020 Ranking System described in previous study was utilized to rank the 15 tested cultivars (Parsons et al., 2020):

(6) $$X_{h}=\sqrt{\sum_{i=1}^{m}{\left(\frac{y_{i,h}}{y_{i,m\,a\,x}}-1\right)}^{2}\,I_{i}\,\left(\sigma_{i,h}/\sigma_{i,m\,a\,x}\right)}$$

The final rank of each cultivar, *X_h*, was determined by the summation of individually determined trait values calculated through trait performance relative to the tested population, ${\left(\frac{y_{i,h}}{y_{i,m\,a\,x}}-1\right)}^{2}$, the trait’s economic importance, *I_i*, and the cultivar’s relative uniformity of trait values compared to the other tested lines, (σ_*i*,*h*_/σ_*i*,*max*_). Economic weights (*I_i*) were determined on an increasing 0–1 continuous scale paralleling consumer and producer concern for trait performance. Weights were determined to be “0.90,” “0.90,” “0.90,” “0.85,” “0.80,” and “0.55,” respectively, for protein-bound lysine content (g/100 g) and traits “Yield,” “Expansion Volume,” “OCFSI,” “Popability,” and “Vitreousness.” Plant height, number of ears per plant, ear length, and flake morphology were considered concurrent to the ranking system results for ultimate selection of best QPP hybrids.

## Results

### Breeding and Selection of BC_3_F_4_ QPP Inbred and Hybrid Cultivars

**FIGURE 1:**
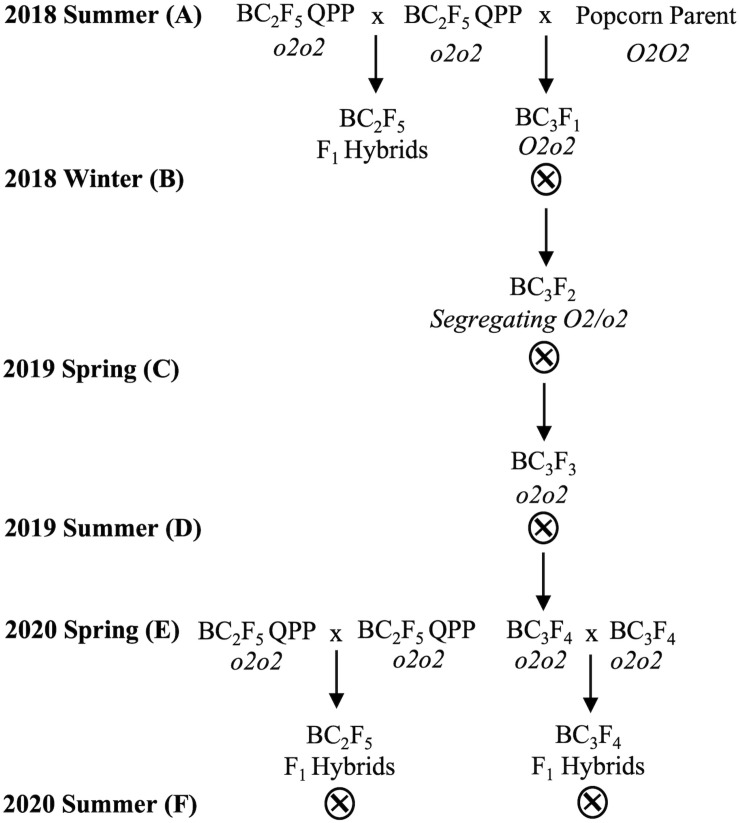
Breeding scheme to produce BC_2_F_5_ and BC_3_F_4_ QPP F_1_ hybrids. Overall breeding scheme from 2018 to 2020. **(A)** In the summer of 2018, BC_2_F_5_ QPP inbreds were crossed in full diallel to produce F_1_ hybrids. BC_2_F_5_ inbreds were also selectively crossed to their respective original popcorn parents to produce heterozygous *O_2_o_2_* BC_3_F_1_ offspring. **(B)** Heterozygotes were self-pollinated to produce segregating BC_3_F_2_ offspring which was selected at the seed based on *opaque-2* phenotyping of vitreousness, protein-profiling, and later marker-assisted selection. **(C)** Homozygous F_2_ seed was grown and self-pollinated prior to 2019 summer. **(D)** Homozygous mutant *o_2_o_2_* BC_3_F_3_ seed was harvested and grown to produce BC_3_F_4_ QPP seed in the summer of 2019. All inbred lines were identified as *o2-*carrying predominantly through protein-profiling. **(E)** BC_2_F_5_ and BC_3_F_4_ QPP inbred lines were grown in the spring of 2020 and selectively crossed to produce similar QPP hybrids of differing backcross generations. **(F)** BC_2_F_5_- and BC_3_F_4_-derived F_1_ hybrids were grown in three locations and evaluated alongside ConAgra elite varieties for selection.

Vitreous BC_3_F_4_ QPP inbred lines were obtained by generational phenotypic and genotypic selection of vitreousness and the *opaque-2* allele, respectively (Figure 1 and Supplementary Figure 1). Homozygous *o2o2* BC_3_F_2_ seedlings were selected in the spring of 2019 and self-pollinated until the BC_3_F_4_ generation. *o2-*induced zein downregulation and non-zein upregulation in BC_3_F_4_ inbred seed were verified through protein extraction and SDS-PAGE, and homozygous allelic introgression of *opaque-2* was verified through marker-assisted selection (not shown). Since BC_3_F_4_ inbred lines were achieved through the backcross of BC_2_F_5_ QPP with original popcorn parents, improved vitreousness of BC_3_ cultivars compared to their BC_2_ counterpart was not attainable (Supplementary Figure 1). Notably, QPP Inbred 3 differed in endosperm color between BC_2_F_5_ and BC_3_F_4_ lines, and QPP BC_3_F_4_ Inbred 3 gained cap opacity. All other QPP inbred lines maintained the same observable level of vitreousness between backcrossing generations (Supplementary Figure 1). Equation 3 estimates from test weight and moisture content revealed a decreased seed size in BC_3_F_4_-derived QPP hybrids compared to BC_2_F_5_ hybrids, and original popcorn parental hybrids had significantly smaller seeds than both QPP populations.

### Phenotypic and Quantitative Assessment of *opaque-2* Initiated Proteomic Rebalancing in Quality Protein Popcorn Hybrids

**FIGURE 2:**
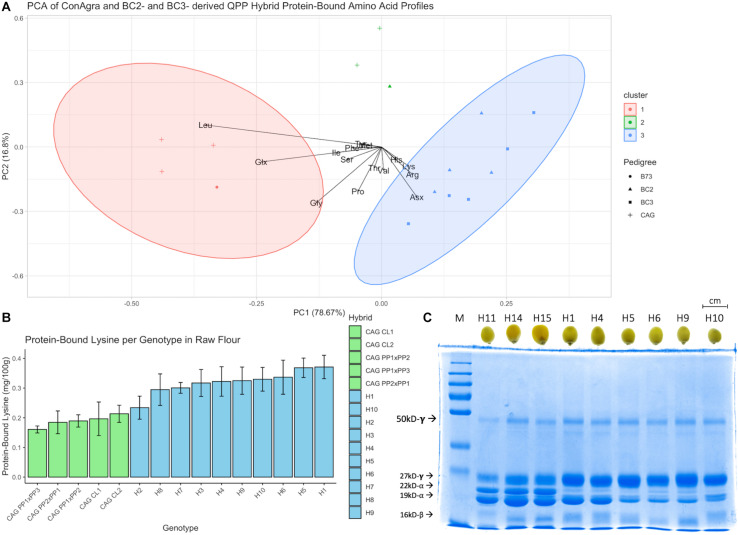
Protein profiling of QPP and ConAgra elite lines. **(A)** Principle component analysis (PCA) of protein-bound amino acids in raw flour of ConAgra elite lines, B73 for reference, and QPP hybrids revealed a distinct segregation between QPP and original popcorn-derived cultivars. B73 grouped with ConAgra lines H12, H13, and H14, while H11 and H15 independently segregated with QPP H2. QPP H2 had a distinct proteome compared to all other QPP hybrids and similar lysine levels compared to ConAgra lines. **(B)** Protein-bound lysine in raw flour of all genotypes revealed significantly higher lysine levels in all QPP lines except H2. **(C)** Zein extraction and SDS-PAGE analysis of randomly selected kernels revealed a significant reduction in 22-kD-alpha zein, varying production of 19-kD-alpha zein, and increased expression of the 27-kD γ-zein in QPP lines compared to ConAgra lines, consistent with a homozygous *opaque-2* profile. Compositely, these results verified the successful introgression and stabilization of the homozygous mutation in the BC_2_F_5_ and BC_3_F_4_ QPP populations.

A random assortment of F_2_ kernels from original popcorn parental crosses, QPP BC_2_F_5_ crosses, and QPP BC_3_F_4_ crosses was obtained for zein and non-zein protein extraction and free and protein-bound amino acid profiling (Figure 2). The first two components in principle component analysis of protein-bound amino acid profiles accounted for 95.47% of variation and clearly separated ConAgra hybrids from QPP hybrids (Figure 2A). A general increase in lysine, arginine, and aspartate/asparagine in QPP hybrids markedly differentiated their cluster from leucine, glycine, and glutamate/glutamine-rich ConAgra hybrids H12, H13, and H14 and B73. According to the nomenclature in Table 1, the BC_2_-derived “H2” displayed a unique protein-bound amino acid profile compared to the rest of the QPP hybrids, as shown by segregating with two ConAgra hybrids, H11 and H15 (outside both red and blue clusters) (Figure 2A). Consistent with these profiling results, H2 had the least amount of protein-bound lysine compared to all QPP hybrids (Figure 2B). Taken as an average, ConAgra hybrids conferred 0.189 ± 0.02 g/100 g of protein-bound lysine, while QPP hybrids presented a 1.7-fold relative increase in protein-bound lysine and averaged a significantly higher value at 0.320 ± 0.04 g/100 g (Supplementary Tables 2–6). In concordance with these results, SDS-PAGE of extracted zein proteins from three randomly selected ConAgra hybrids, BC_2_F_5_- and BC_3_F_4_-derived QPP hybrids exhibited expected profiles (Figure 2C). ConAgra lines displayed the wild-type zein profile of abundant 22-kD α-zein, relatively downregulated 27-kD γ-zein, and variable 19-kD α-zein (Figure 2C). All six QPP hybrids had 22-kD α-zein as specified by *opaque-2*, 19-kD α-zein variability, and high 27-kD γ-zein resulting from the duplication which is responsible for the improved vitreousness (Figure 2C). Interestingly, H1 and H4 displayed a visible increase in 19-kD α-zein abundance that was not seen in the BC_3_ counterparts H6 and H9, respectively.

### Distinction in Agronomic Trait Performance Between QPP BC_2_F_5_, BC_3_F_4_, and Popcorn Parental Hybrids

Yield Eq. 2 offered a yield estimate in bushels/acre at 15.5% moisture, a common unit to evaluate maize yields, and values were converted into kilograms/square meters. ConAgra Commercial Line 2 (H15) was grown as a high-yielding target with average popping traits, while ConAgra Commercial Line 1 (H14) was grown and evaluated for its premier popping characteristics and average yield (Table 1). H15 exhibited the maximum yield average at 0.563 kg/m^2^ (89.53 bu/ac), while H14 yielded 0.428 kg/m^2^ (68.07 bu/ac; Table 2). On average, BC_2_-derived QPP hybrids yields were not significantly different from ConAgra lines with ∼0.390 kg/m^2^ (62 bu/ac) and ∼0.421 kg/m^2^ (67 bu/ac) yields, respectively (Table 2). BC_3_-derived hybrids yielded an average of 0.333 kg/m^2^ (53 bu/ac), significantly lower than the other two groups. Specifically comparing H15 to all QPP and ConAgra hybrids, only QPP H5 had no significantly different yield measure. Conversely, all QPP hybrids except H6, H7, and H8 conferred comparable yields to H14.

**TABLE 2:** Select trait measurements of cultivars tested in 2020 summer trials.

Cultivar	Ref. No.	Yield 1		Yield 2		PB lysine (raw flour)		Expansion volume		OCFSI		Popability		Vitreousness	
								
		*Grams dry matter/m^2^ (lbs/ft^2^)*	*sd*	*15.5% moisture kg/m^2^ (bushel/ac)*	*sd*	*g/100g*	*sd*	*cc per gram*	*sd*	*unit*	*sd*	*%*	*sd*	*1-7 scale*	*sd*
BC_2_F_5_ F_1_ Hybrid	**H1**	312.48^cd^ (0.064)	*63.47 (0.013)*	0.3934^cde^ (62.54)	*0.106 (16.82)*	0.371^*a*^	*0.039*	26.11^*efg*^	*3.98*	3.53^*d*^	*0.62*	96.37^*fgh*^	*1.00*	5.56^*bc*^	*0.92*
	**H2**	273.42^cd^ (0.056)	*73.24 (0.015)*	0.3560^de^ (56.59)	*0.085 (13.43)*	0.234^*bcdef*^	*0.039*	19.11^*j*^	*3.76*	2.56^*f*^	*0.44*	95.49^*hi*^	*1.29*	4.75^*cde*^	*1.10*
	**H3**	273.42^cd^ (0.056)	*58.59 (0.012)*	0.3388^de^ (53.86)	*0.092 (14.65)*	0.317^*abcd*^	*0.045*	24.22^*ghi*^	*2.64*	3.14^*de*^	*0.20*	96.64^*efg*^	*1.31*	5.33^*bcd*^	*0.25*
	**H4**	273.42^cd^ (0.056)	*48.83 (0.010)*	0.3532^de^ (56.15)	*0.073 (11.56)*	0.322^*abc*^	*0.050*	25.00^*fghi*^	*3.61*	3.42^*d*^	*0.38*	96.49^*efgh*^	*1.25*	6.22^*ab*^	*0.83*
	**H5**	400.37^ab^ (0.082)	*63.47 (0.013)*	0.5038^ab^ (81.10)	*0.109 (17.30)*	0.368^*a*^	*0.033*	19.56^*j*^	*4.42*	2.68^*ef*^	*0.67*	94.84^*i*^	*1.23*	4.64^*de*^	*0.78*
*average*		306.24^*b*^ (0.063)	*77.124 (0.016)*	0.389^a^ (61.848)	*0.108 (17.192)*	0.323^*a*^	*0.062*	22.8^*c*^	*4.61*	3.06^*b*^	*0.614*	95.97^*c*^	*1.36*	5.30^*b*^	*0.98*
BC_3_F_4_ F_1_ Hybrid	**H6**	244.13^d^ (0.050)	*58.59 (0.012)*	0.3091^e^ (49.14)	*0.081 (12.93)*	0.337^*ab*^	*0.057*	27.78^*ef*^	*3.70*	3.36^*d*^	*0.54*	97.57^*bcde*^	*1.23*	6.47^*a*^	*0.54*
	**H7**	253.89^d^ (0.052)	*53.71 (0.011)*	0.3105^e^ (49.37)	*0.073 (11.62)*	0.300^*abcde*^	*0.018*	21.67^*ij*^	*2.06*	2.74^*ef*^	*0.26*	96.89^*defg*^	*0.98*	3.94^*e*^	*0.85*
	**H8**	244.13^d^ (0.050)	*53.71 (0.011)*	0.3072^e^ (48.84)	*0.085 (13.53)*	0.295^*abcde*^	*0.053*	25.33^*fgh*^	*3.87*	3.15^*de*^	*0.46*	97.22^*cdef*^	*0.93*	5.06^*cd*^	*0.81*
	**H9**	273.42^cd^ (0.056)	*87.89 (0.018)*	0.3391^de^ (53.92)	*0.130 (20.66)*	0.325^*abc*^	*0.046*	29.44^*de*^	*4.98*	3.38^*d*^	*0.38*	97.95^*bcd*^	*0.97*	5.56^*bc*^	*1.31*
	**H10**	317.36^cd^ (0.065)	*39.06 (0.008)*	0.3901^cde^ (62.01)	*0.065 (10.33)*	0.330^*abc*^	*0.040*	22.22^*hij*^	*2.95*	2.81^*ef*^	*0.16*	96.03^*gh*^	*1.18*	5.44^*bcd*^	*1.10*
*average*		265.56^c^ (0.054)	*63.37 (0.013)*	0.331^b^ (52.657)	*0.091 (14.533)*	0.317^*a*^	*0.042*	25.29^*b*^	*4.63*	3.09^b^	*0.457*	97.13^*b*^	*1.21*	5.29^b^	*1.23*
ConAgra^®^ Brands Popcorn	**H11**	283.19^cd^ (0.058)	*48.83 (0.010)*	0.3459^de^ (54.99)	*0.106 (16.81)*	0.190^ef^	*0.021*	34.44^bc^	*4.67*	4.63^*bc*^	*0.48*	98.12^abc^	*1.19*	7.00^a^	*0.00*
	**H12**	253.89^d^ (0.052)	*68.36 (0.014)*	0.3107^e^ (49.39)	*0.076 (12.07)*	0.185^ef^	*0.038*	33.22^c^	*4.09*	4.32^c^	*0.77*	97.93^bcd^	*1.00*	6.72^a^	*0.83*
	**H13**	390.60^ab^ (0.080)	*78.12 (0.016)*	0.4693^bc^ (74.61)	*0.103 (16.31)*	0.161^f^	*0.012*	37.33^*ab*^	*5.29*	4.77^bc^	*0.71*	98.64^ab^	*0.89*	7.00^*a*^	*0.00*
	**H14**	346.66^bc^ (0.071)	*19.53 (0.004)*	0.4282^bcd^ (68.07)	*0.044 (7.05)*	0.197^def^	*0.057*	39.89^*a*^	*3.79*	5.52^a^	*0.85*	99.16^a^	*0.23*	7.00^*a*^	*0.00*
	**H15**	458.96^a^ (0.094)	*97.65 (0.020)*	0.5632^a^ (89.53)	*0.156 (24.73)*	0.214^cdef^	*0.029*	32.00^*cd*^	*5.17*	4.93^*b*^	*0.37*	98.22^abc^	*1.24*	7.00^*a*^	*0.00*
*average*		346.17^a^ (0.071)	*98.67 (0.020)*	0.423^a^ (67.32)	*0.134 (21.338)*	0.189^*b*^	*0.034*	35.38^a^	*5.29*	4.83^*a*^	*0.748*	98.41^a^	*1.03*	6.94^a^	*0.37*

*Trait values and standard deviations that were utilized for the 2020 Ranking System (except for Yield 2) are shown. Significance between cultivar measurements is indicated by lettered superscripts.*

All QPP hybrids were not significantly different in yield compared to their respective popcorn parental pedigrees except H2 and H7, two hybrids stemming out of the same, H13-equivalent, popcorn pedigree (Tables 1, 2).

Plant height, ear length, and number of ears per plant were measured prior to combine harvesting but low, insignificant correlations were found between hand measured traits and yield estimates (correlations not shown; Supplementary Table 1). Therefore, hand measured traits were not considered in the overall ranking of hybrids using the 2020 Ranking System.

### Popping Quality Trait Evaluation Between ConAgra Elite Hybrids and Differing QPP Backcross-Generated Hybrids

Expansion volume, OCFSI (Oil-popped Crude FSI, a measure of average flake size), and popability measurements displayed ConAgra varietal advantage compared to all QPP hybrids (Table 2). Percentage of grain moisture was ascertained prior to popping of each sample and had no significant effect on EV. Though the location effect was significant (α0.05), no interactions were visually identified when analyzing the data through backcrossed groups. The Mead location resulted in higher percentages of grain damage/mold, and a percentage of mold was noted per each EU. H2 experienced 50% mold damage per sample, while all other QPP and ConAgra hybrids did not have significantly different levels of damage. After popping, ConAgra hybrids averaged an EV of 35.38 ± 5.29 cubic centimeters/gram, BC_2_-derived QPP hybrids averaged 22.8 ± 4.6 cubic centimeters/gram, and BC_3_-derived QPP hybrids averaged 25.28 ± 4.63 cubic centimeters/gram, demonstrating significant differences between all groups and a significant improvement in EV after the third QPP backcross (Table 2). Comparing QPP hybrids with commercial lines H14 and H15, H9 was not significantly different in EV measure compared to H15.

In terms of popability (percentage of kernels that pop), H9 did not show a significant difference compared to H11 (its corresponding ConAgra hybrid in pedigree) and H15. H6 and H8 also did not display significantly different popping values compared to their ConAgra-related hybrids (H12 and H11, respectively) and H15. Categorizing hybrids into backcross groups and ConAgra^®^ controls rendered significant differences between all three groups (Table 2). QPP BC_2_-derived hybrids showed the lowest popability percentage at 96%, while BC_3_-derived hybrid and ConAgra^®^ hybrids were narrowly higher with averages of 97.1 and 98.4%, respectively (Table 2).

Oil Crude FSI values showed insignificant differences between backcrossing generations, but ConAgra hybrids did have a significantly higher FSI compared to QPP hybrids (Table 2). All OCFSI averages ranged from 2.56 to 5.52, with H2 holding the lowest value and H14 holding the highest (Table 2).

An overview of these popping trait values identified trends between QPP hybrids, backcrossing groups, and ConAgra-respective hybrids. H2 and H5 had the lowest EV and OCFSI values out of all QPP hybrids, followed by their BC_3_-counterparts H7 and H10. All four of these hybrids consistently had the lowest averages for all three popping traits compared to all other tested hybrids, though the BC_3_ hybrids did have significantly higher popability values compared to the respective BC_2_ varieties. These four QPP hybrids were also derived from the same PP1 x PP3 (H13) ConAgra pedigree, which did not have correspondingly lower popping quality trait values compared to the other ConAgra varieties (Table 2).

Quality Protein Popcorn hybrids that noticeably performed higher than average on popping quality traits were H1, H6, and H9 (Table 2). These three hybrids had the highest EV measurements, H6 and H9 had the highest popability percentages, and the trio had the highest OCFSI measurements accompanied by H4 (Table 2). H12, the corresponding ConAgra hybrid to H1 and H6, held the lowest OCFSI, lowest popability, and second lowest EV measurements compared to other ConAgra hybrids.

### Flake Morphology Assessment of Tested Hybrids

**FIGURE 3:**
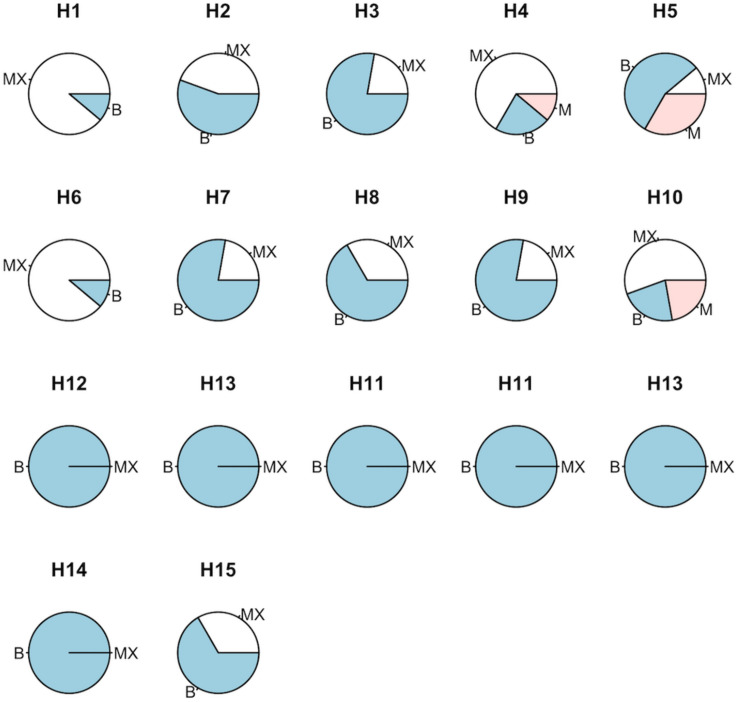
Flake morphology assessment of QPP and ConAgra elite lines. Random samples of QPP and ConAgra lines from each experimental plot were given a description of butterfly (B—blue), mushroom (M—red), or mixed flake morphology (MX—white). Each cultivar was assigned a total of nine descriptions (three from each of the three locations). All ConAgra varieties were assigned butterfly morphology except H15, which was assigned three MX morphologies. QPP BC_2_-derived hybrids are displayed on the first row, respective QPP BC_3_-derived hybrids are on the second row, and original popcorn hybrids from the respective pedigrees are arranged on the third row to enable column comparison between similar QPP and ConAgra pedigrees. Commercial lines H14 and H15 are positioned on the fourth row.

Immediately after popping, flakes were assessed and each EU was categorized into butterfly, mushroom, or mixed morphologies (blue, red, and white, respectively; Figure 3). QPP hybrids derived from corresponding backgrounds but different backcrosses showed mostly similar flake morphology patterns (Figure 3). All ConAgra-derived hybrids (H11–H13) and H14 were attributed unwavering uniformly of “butterfly” morphology. H1 and H6 largely displayed a “mixed” morphology with a single “butterfly” distinction. H2 and H7 both had a majority of butterfly flake morphology assignments. H3, H7, and H9 showed the most uniform butterfly morphology followed by H8. H4, H5, and H10 were assigned varying flake morphologies. H4 and H10 had a majority of mixed flakes, while H5 had a majority “butterfly.” All three of these QPP hybrids had at least one distinct “mushroom” assignment. H15 was the only ConAgra line that had an assignment other than “butterfly” in that three EUs were categorized as “mixed” morphology (Figure 3).

### Free and Protein-Bound Lysine in QPP Compared to Parental Popcorn Hybrids in Raw and Air-Popped Forms

As previously stated, QPP hybrids showed a 1.7-fold increase in protein-bound lysine compared to ConAgra hybrids in the raw flour form (Supplementary Tables 2–6). After popping, protein-bound lysine levels in popped flakes were 1.84-fold higher in QPP hybrids compared to ConAgra hybrids with 0.24 ± 0.04 g/100 g and 0.13 ± 0.01 g/100 g values, respectively (Supplementary Tables 2–6). Lysine values between BC_2_ and BC_3_ backcrossed QPP populations were not significantly different for both protein-bound and free lysine in both ground and air-popped forms (α0.05). Air popping decreased protein-bound lysine levels by ∼30.3% in all hybrids with a significant Pearson’s correlation coefficient of 0.872(α0.05). However, H4 presented insignificant changes in protein-bound lysine content before and after popping likely due to sample preparation error. Excluding H4 data from the correlation test rendered a significant Pearson’s correlation coefficient of 0.948 (α0.05) between raw flour protein-bound lysine and air-popped protein bound lysine levels. Moreover, despite the 30% decrease in lysine, air-popped QPP hybrids still had higher protein-bound lysine levels than ConAgra lines in the raw flour form (Supplementary Tables 2–6).

An insignificant reduction differential after popping between ConAgra and QPP hybrids in both protein-bound and free lysine was found. Free lysine levels decreased after popping by roughly 20% in all cultivars though values correlated with a Pearson’s coefficient of 0.746 (Supplementary Tables 7–10). Free lysine levels were minimal compared to protein-bound levels, rendering an average of 0.0014 ± 0.0003 g/100 g lysine in ConAgra hybrids and 0.0071 ± 0.003 g/100 g in QPP hybrids in the raw flour form (Supplementary Figure 2). These averages indicate QPP hybrids conferred a 4.95-fold relative increase in free lysine levels in raw flour and a 5.44-fold relative increase in free lysine retained after popping, with averages of 0.00519 and 0.00095 g/100 g in QPP and ConAgra hybrids, respectively. Though these large fold-increases in free lysine were significant, free lysine in the air-popped samples only accounted for ∼2 and ∼0.7% of the total lysine in QPP and ConAgra hybrid popped flakes, respectively (Supplementary Tables 7–10).

Specifically comparing lysine levels between ConAgra commercial lines H14 and H15 and QPP hybrids, QPP H1, H4, H5, H6, H9, and H10 all had significantly higher protein-bound lysine levels in the raw form than H14, and H1, H5, and H6 held significantly higher levels than H15 (Figure 2B and Supplementary Tables 7–10). In the popped form, all QPP hybrids except H2 displayed significantly higher protein-bound lysine levels than both H14 and H15, indicating a significantly higher lysine intake in the consumable form. Overall, QPP hybrids showed higher levels of lysine in the ground kernels and popped flakes compared to ConAgra’s currently commercialized popcorn cultivars.

### 2020 Ranking System: Evaluation and Ranking of Hybrids

**FIGURE 4:**
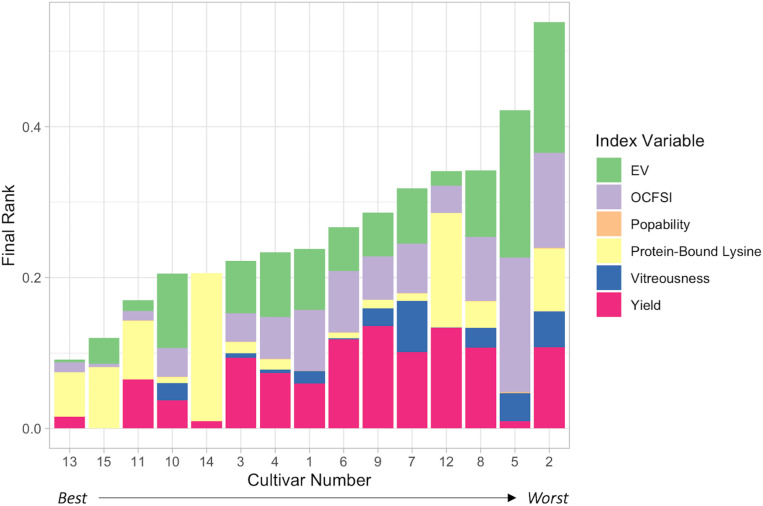
2020 Ranking System Selection Index Results. Utilization of the 2020 Ranking System enabled a visual display of overall cultivar ranking from best to worst, left to right, respectively. Color by variable identified individual hybrid pitfalls (the longer the stacked column, the farther from the best hybrid) and high trait values. H13, PP1 x PP3, ranked highest out of all hybrids, scoring relatively lower only because of its lack of protein-bound lysine content. QPP H10 ranked the best compared to all other QPP lines and ranked higher than H14, or Commercial Line 1. QPP BC_2_-derived hybrids H3, H4, and H1 ranked, respectively, higher than the rest, while BC_3_-derived hybrids H6, H9, and H7 all ranked higher than original popcorn hybrid line H12. H8, H5, and H2 ranked lowest out of all hybrids. Economic weights for each trait were determined to reflect consumer and producer interests in a ready-to-eat Quality Protein Popcorn product. Lysine levels, yield, and expansion volume were considered equally important traits while ranking all hybrids.

Economic weights “0.90,” “0.90,” “0.90,” “0.85,” “0.80,” and “0.55,” respectively, for protein-bound lysine content (g/100 g), Yield (Eq. 1), EV, OCFSI, popability, and vitreousness were utilized in the 2020 Ranking System (Table 3, Figure 4, and Supplementary Table 11). H13 had the best, lowest ranking due to its above average measurements in all traits except for protein-bound lysine content. H15 ranked second due to its relatively lower EV compared to other ConAgra hybrids. H11 ranked third in part due to its poorer yield, and H12 ranked very low due to below average yield and popping traits. QPP BC_3_-derived hybrid H10 ranked fourth overall despite its poor popping quality traits, followed by H14, H3, H4, H1, H6, and H9. H5 was ranked second lowest due to very poor popping traits, and H2 was ranked last due to low yields, popping traits, and relatively lower lysine abundance (Figure 4). Overall, most ConAgra^®^ hybrids ranked higher than most QPP cultivars; however, H10, H3, H4, and H1 showed close ranking values compared to commercial hybrids H15 and H14 (Figure 4).

**TABLE 3:** Economic Values assigned for traits in 2020 Ranking System.

Trait	Selection index economic value (*I*_*i*_)
Protein-bound lysine (g/100 g)	0.90
Yield (lbs/ft^2^)	0.90
Expansion volume (cc/g)	0.90
OCFSI	0.85
Popability (%)	0.80
Vitreousness	0.55

*Economic weighting values were determined to best reflect producer and consumer concern in a theoretical QPP, ready-to-eat product. Protein-bound lysine, yield, and expansion volume were each considered equally important traits during selection, while OCFSI, popability, and vitreousness were, respectively, given lesser importance for overall selection due to their relationships with the three key traits.*

## Discussion

### QPP Backcross Breeding and Selection

The production of BC_2_F_5_ QPP inbred lines with highly vitreous endosperm, high lysine content, and restored popping characteristics offered scope for successful popcorn hybrid production utilizing dent maize germplasm. However, due to the temporary loss of popping capability in the early breeding stages of QPP and restoration in the final stage, popping traits such as expansion volume, popability, and OCFSI were not selected for during inbred production and final determination of elite QPP inbreds (Ren et al., 2018). Moreover, a preliminary popping test of selected inbreds identified overall reduced expansion volume with variability. After initial hybridization of inbred lines and selection of 44 BC_2_F_5_-derived QPP hybrids, popping traits were analyzed and found to be significantly, moderately lower than original popcorn parental lines (Parsons et al., 2020).

Previous studies have postulated that popping expansion, the premier quality trait of popcorn, is predominantly a highly heritable additive trait regulated by three to five major genes (Dofing et al., 1991; Pereira and Amaral Júnior, 2001; Ziegler, 2001; Li et al., 2003; Coan et al., 2019). A recent crossing study aimed at studying the mode of expansion volume inheritance found that one backcross to the original popcorn parental line recovered 75% of the popping expansion of the original parent, and the BC_2_-cross was not produced or tested (Coan et al., 2019). Indeed, previous inheritance-centered studies agree that a single backcross is sufficient for recovering a majority of popping capacity fit for genetic studies, but not enough to achieve synonymous popping trait measurements to the original parent (Li et al., 2003, 2007, 2008). Dating back to 1949, Crumbacker et al. postulated that two backcross generations to the original popcorn parent were sufficient for recovering popping expansion volume after a dent by popcorn cross, and limited, but recent studies have validated this approach (Crumbaker et al., 1949; Li et al., 2004; Niu et al., 2008).

Though the theoretical genomic recovery of the recurrent parent in a BC_2_ cross is 0.875, and 0.9375 for a BC_3_ backcross, these proportions do not consider genomic or phenotypic selection measures employed throughout a breeding program (Collard et al., 2005; Uptmoor et al., 2006; Silva et al., 2007; Gupta et al., 2010; Ramos et al., 2011). One study converting two non-QPM dent lines into QPM found that the selected BC_2_ lines recovered an average of 0.901–0.972 of the recurrent parental genome, and the BC_3_ generation recovered 0.971–0.996 utilizing foreground selection (Thakur et al., 2014). This breeding program utilized two dent maize parents rather than a popcorn recurrent and dent maize donor parent, and solely the QPM *opaque-2* allele and required modifiers were selected. Considering the current study’s aim to select for QPM-based amino acid and endosperm modifier genes and popcorn-based phenotypic traits such as seed size, kernel morphology, and popping traits—all of which have uncertain genetic locations—the genetic contribution of both parents in the QPP BC_2_F_5_ inbred lines could not be predicted as theoretically distributed nor necessarily favoring the recurrent parent to such an extent as found by the previous QPM-conversion study. Moreover, without knowledge of the location for necessary loci from both the recurrent and donor parents, sequencing the few QPP lines available would have provided genetic contribution proportions but would do little to aid in identifying premier inbreds or popping or QPM trait quantitative trait loci due to the limited number of lines available. Therefore, as previous studies have attributed popping trait improvement of dent by popcorn crosses to backcross-based breeding methods, and theoretical genetic contribution of the recurrent parent could be increased by 6.25% by an additional backcross, BC_2_F_5_ QPP inbred lines were crossed to the original popcorn parents and self-pollinated and selected to the F_4_ generation. Given the availability for heterozygosity in the initial BC_3_ cross was only ∼0.0625, three generations of self-pollinating and selection rendered the availability for heterozygosity in the BC_3_F_4_ lines at 0.39%, or the equivalent to an F_8_ generation without backcrossing (Semagn et al., 2006; Gupta et al., 2010).

Though the theoretical additional genetic contribution by the recurrent popcorn parental parent was 0.0625 between the BC_2_ and BC_3_ generations, empirical studies sequencing backcross population of various plants do indicate high variability between backcross populations and rather unpredictable genetic proportions (Uptmoor et al., 2006; Ramos et al., 2011; Thakur et al., 2014). Thus, without sequencing BC_2_F_5_ and BC_3_F_4_ inbred lines, the extent and location of selected QPM and popcorn loci, and the final genetic contributions of both parents, remain unknown. Future dent by popcorn breeding may benefit more profitably by backcrossing after genetic locations of popcorn traits and QPM endosperm-restorer and amino acid modifier genes have been identified. Previous and current work have suggested genetic whereabouts for both popping traits and *opaque-2* related genes, but the elucidation of exact locations coupled to available genetic markers remains unavailable (Holding et al., 2008, 2011; Li et al., 2009; Gutiérrez-Rojas et al., 2010; Wu et al., 2010; Babu et al., 2015; Pandey et al., 2015; Coan et al., 2019; Senhorinho et al., 2019). The potential for verified markers in both suites of genes coupled to the declining cost of genomic sequencing offers scope for future dent by popcorn breeding systems that aim to improve agronomics within popcorn cultivars while maintaining synonymous popping characteristics.

### Simultaneous Comparisons Between Backcrossed Generations and ConAgra Elite Lines

Rapid breeding of the BC_3_F_4_ QPP inbred lines enabled simultaneous comparison between the BC_2_- and BC_3_-derived hybrids and between all QPP lines and ConAgra elite cultivars. The kernel mold damage experienced at the Mead, NE location gave opportunity to test pest susceptibilities between BC_2_-, BC_3_-, and non-QPM popcorn lines. Initial introgression of *opaque-2* without necessary endosperm modifiers into various dent maize lines resulted in inferior agronomics and higher pest/rot susceptibility (Prasanna et al., 2001). Other than H2, a QPP hybrid inferior in all other evaluated traits, all QPP lines did not experience significantly different mold susceptibility compared to ConAgra varieties. These results suggest the successful introgression of original dent allele *opaque-2* and essential endosperm modifiers into a popcorn background. Comparing QPP backcross populations, results indicated that an additional popcorn backcross slightly improved QPP popping characteristics compared to BC_2_-derived hybrids; however, average QPP popping traits were still significantly lower than ConAgra lines. Average BC_2_ hybrid expansion volume measurements were roughly 64% of ConAgra volumes, while BC_3_ hybrids held 71% of premier volume values. OCFSI values showed similar ratios between QPP and ConAgra lines, while popability measurements were similar between all hybrids. The discrepancy between previously published backcross-restored popping traits and QPP inbreds is likely due to the selection measures imposed during inbreeding (Coan et al., 2019). Without known locations and extent of required QPM dent maize loci introgression, and with known repulsion phase linkages between yield and expansion volume, and with inherent selection of agronomic characteristics throughout QPP inbred line production, unintentional selection against expansion volume could have been employed (Sprague and Dudley, 1988; Dofing et al., 1991; Ziegler and Ashman, 1994; Ren et al., 2018).

Despite not attaining synonymous popping characteristics after an additional backcross to the original parents, BC_3_-derived lines displayed significant improvements in these traits compared to BC_2_-derived lines. However, the trade-off between popping and agronomic characteristics was apparent as BC_2_-derived lines had significantly better yield averages. Therefore, utilization of the previously published 2020 Ranking System equation proved helpful in holistically discriminating between BC_2_- and BC_3_-derived hybrids and comparing them individually to original popcorn lines (Parsons et al., 2020). In this analysis, a machine-harvested yield estimate took the place of previously used hand-measured agronomic traits. Additionally, robust popping trait analysis enabled a more precise measure for popping quality compared to the previous analysis which allowed vitreousness to be given a lesser weight in the present selection (Parsons et al., 2020). Lysine content was introduced into the equation to impart equal weight between agronomics, popping, and quality protein traits. Therefore, protein-bound endosperm lysine content, yield, and expansion volume were considered equally paramount in the final selection of QPP hybrids and were each given an economic weight of 0.90. Flake size and popability measures were also considered in final rankings to highlight premier popping in QPP, but these traits were given relatively lesser influence through reduced economic weights. Vitreousness had the least influence on overall ranking but still imparted value in the ranking system due to its relationship with all premier traits (lysine, agronomics, and popping). Final ranking identified top QPP hybrids as H10, H3, H4, H1, H6, and H9, in respective order. Though the highest ranked hybrid was a BC_3_-derived cross, BC_2_-crosses H3, H4, and H1 were superior to BC_3_-crosses H6 and H9. These results suggest that the third-backcrossed population did not produce satisfactory popping results to warrant the time, assets, and effort allotted to producing it. However, the significant improvements in BC_3_-derived hybrids H7 and H10 compared to their BC_2_ counterparts H2 and H5, respectively, show specific potential in this breeding scheme if genetic selection could be conducted more specifically. Overall, the six most elite hybrids stemmed equally from the BC_2_ population and the BC_3_ population which rendered a diverse set of potentially marketable QPP varieties fit for consideration.

### Flake Morphology of Selected QPP Hybrids

All QPP hybrids exhibited varying mixtures of butterfly and mushroom flake morphologies. H3, H7, and H9 demonstrated the closest resemblance to their ConAgra respective hybrids, followed by H2 and H8. H1 and H6, hybrids from the same QPP cross but of differing backcrossed generations, exhibited the same morphological behavior in mostly a mixture of flakes. H4 and H9 differed most dramatically between backcrosses in this trait. H9 showed a majority of butterfly flakes, while H4 had a majority mixture, followed by some samples popping solely butterfly and one sample popping solely mushroom. This morphological profile was similarly mirrored by H10, though H10 had one more sample labeled “mushroom” rather than a mixture. H5 interestingly only had one mixed sample; the rest popped either solely butterfly or solely mushroom. The location effect on these particular hybrids’ popping morphology was significant (Supplementary Table 11). Out of the nine samples analyzed, the three H5 samples taken from Lincoln, NE were considered “butterfly,” followed by the secondary location rendering two butterfly samples and one mushroom sample, and finally the Colby, KS location had two mushroom samples and one mixed sample. Similar to H5, H10 had three “mixed” samples at Lincoln, NE, followed by two butterfly samples and one mixed sample at Mead, NE, and two mushroom and one mixed sample taken from Colby, KS. Previous studies analyzing the environmental effect on popcorn flake morphology are limited, but one study in 2012 identified growing location as a significant factor in popcorn flake morphology though the extent of locational influence on morphology in comparison to other intrinsic and external factors remained elusive (Sweley et al., 2012). The narrow number of hybrids and samples tested per location limited these results’ identification of particular flake morphological responses to certain environmental influences; however, like the 2012 study, the locational effect on flake morphology was found to be significant and warrants consideration when typifying future popcorn varietal flake morphologies.

### QPP Cultivars Exhibit Elevated Lysine Levels Compared to ConAgra Elite Lines in Flour From Raw and Air-Popped Kernels

Previous studies have shown that tryptophan and lysine levels within the same maize variety positively correlate in relative abundance in the zein fraction and thus in the entire endosperm (Hernandez and Bates, 1969; Krivanek et al., 2007; Olakojo et al., 2007; Ren et al., 2018; Parsons et al., 2020). Due to acidic hydrolysis’ destruction of protein-bound tryptophan, lysine levels were recovered and used as a benchmark for *opaque-2-*derived lysine and tryptophan increases compared to ConAgra varieties (Angelovici et al., 2013; Yobi and Angelovici, 2018). Protein-bound lysine levels in raw flour displayed a significant difference between ConAgra varieties and QPP cultivars, and no significant difference was found between BC_2_ and BC_3_ derived QPP cultivars. On average, QPP varieties conferred 0.320 ± 0.039 and ConAgra cultivars held 0.189 ± 0.019 g/100 g protein-bound lysine in the raw flour, respectively. After popping, lysine levels decreased by ∼30% to 0.235 ± 0.042 g/100 g lysine and 0.128 ± 0.006 g/100g lysine in QPP and ConAgra cultivars, respectively. Even after air-popping, QPP cultivars retained more lysine than what was produced in non-QPM popcorn raw kernel flour.

Previous analysis of QPP and non-QPP popcorn lysine content revealed a slightly higher protein-bound lysine level than the current study indicates (Parsons et al., 2020). However, considering the ratio between non-QPM and QPM popcorn lysine levels is consistent between analyses, these results compositely suggest a stable and reliable increase in lysine content in air-popped QPP varieties compared to currently marketed popcorn. Contextually, a 68 kg (150 pound) individual is generally recommended to ingest 2.108 g of lysine per day (Elango et al., 2009). These results suggest that the equivalent of one microwavable bag of QPP air-popped popcorn (∼47 g) would fulfill 5.2% of this daily lysine requirement as opposed to a 2.8% fulfillment available through currently commercialized popcorn varieties.

### Conclusion: Final Selection of QPP Hybrids

The holistic evaluation of QPP hybrids with ConAgra controls allowed for the fulfillment of the first objective of this study, the simultaneous comparison of BC_2_F_5_ and BC_3_F_4_ genetic backgrounds with ConAgra elite lines to further select QPP best fit for potential commercialization. This evaluation found the BC_3_ hybrids had significantly lower yields compared to both ConAgra and BC_2_ groups, but the BC_3_ cultivars had significantly improved popping traits compared to the BC_2_ hybrids. In all popping traits evaluated, specifically expansion volume, OCFSI, and popability, all three groups had significantly different averages with ConAgra elite lines leading, followed by BC_3_F_4_-derived QPP hybrids, and lastly BC_2_F_5_*-*derived QPP hybrids. As only two BC_3_-derived lines performed better than their BC_2_-derived counterparts in the final ranking utilizing the 2020 Ranking System, it is uncertain whether the time and resources spent introducing another backcross to this germplasm are justifiable. Thus, this study may evoke caution in further backcrossing for other dent by popcorn breeding programs aimed at improving agronomic and popping quality traits. However, the significant improvement in H7 and H10 compared to H2 and H5 demonstrates success, albeit rather indiscriminate, for this breeding plan. The significant increase in protein-bound lysine in all QPP hybrids except H2 compared to ConAgra elite lines in popped flakes validates the successful introgression of the QPM *opaque-2* allele and necessary endosperm modifier genes for restored popping. Additionally, the PCA of the protein-bound amino acid protein profile clusters all QPP separately from ConAgra lines except H2. H2 performed the worst out of all hybrids in multiple different analyses, holding the lowest protein-bound lysine content, expansion volume, OCFSI, second lowest popability, third lowest vitreousness, and eighth highest measurement in yield. Conversely, QPP hybrids H10, H3, H4, H1, H6, and H9 all showed high lysine values and returned overall higher ranking values compared to the four other QPP hybrids. Utilizing the 2020 Ranking System enabled holistic evaluation of hybrid agronomic, popping quality, and protein quality fitness and enabled final QPP selection. This Ranking System is a transferable and available resource fit for hybrid production and testing programs. The QPP hybrids highlighted from this ranking system displayed sufficient agronomic and popping quality trait evaluations and significantly higher lysine content compared to currently marketed varieties, offering evidence for their potential marketability. Further, recent sensory analysis comparing H15, H14, and six QPP hybrids demonstrated consumer preference for QPP’s increased diversity in taste and texture compared to currently marketed lines. This taste-test identified QPP hybrids H4, H8, and H3 as forerunner cultivars in consumer likability. Taken together, these results suggest that QPP hybrids H4 and H3 are premier candidates for further testing and reasonably fit for commercialization.

## Data Availability Statement

The original contributions presented in the study are included in the article/Supplementary Material, further inquiries can be directed to the corresponding author/s.

## Author Contributions

LP and DH designed the research. YR and DH produced BC_2_F_5_ inbred lines. LP, AY, RA, OR, and DH performed the research. LP, AY, RA, and DH analyzed the data. LP and DH wrote the manuscript. All authors contributed to the article and approved the submitted version.

## Conflict of Interest

The authors declare that this study received funding from ConAgra Brands^®^. Moreover, OR was employed by the company ConAgra Brands^®^. The funder was not involved in the study design, collection, analysis, interpretation of data, the writing of this article, or the decision to submit it for publication. The remaining authors declare that the research was conducted in the absence of any commercial or financial relationships that could be construed as a potential conflict of interest.
