# Supplementary material for: Final Selection of Quality Protein Popcorn Hybrids
Source: Front Plant Sci. 2021 Mar 24;12:658456. doi: 10.3389/fpls.2021.658456 (PMC8025670; doi:10.3389/fpls.2021.658456)
Supplement: Supplementary file 1 [file Data_Sheet_1.PDF]

Supplementary Materials

Frontiers in Plant Science

Parsons et al., 2021

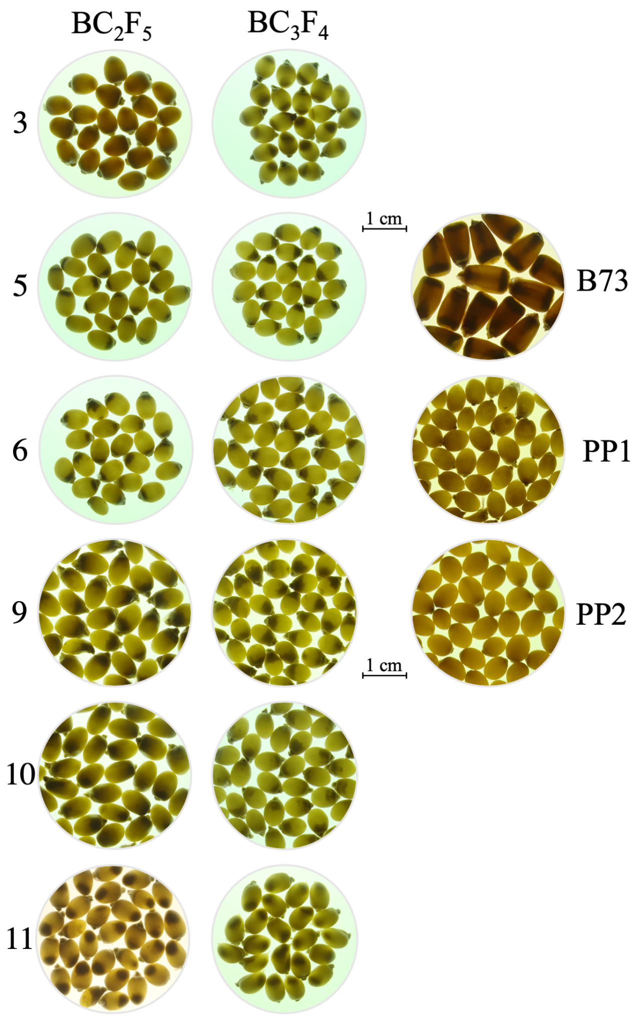

Supplementary Figure 1 | Scaled comparison of BC_2F_5 and BC_3F_4 QPP hybrids and ConAgra Popcorn Parent 1, Popcorn Parent 2, and B73. Overall, BC_3- derived inbreds displayed smaller kernels which produced significantly smaller F_1 hybrid kernels compared to BC_2-derived hybrids, while popcorn parents produced the smallest seed size in non-QPM popcorn hybrids.

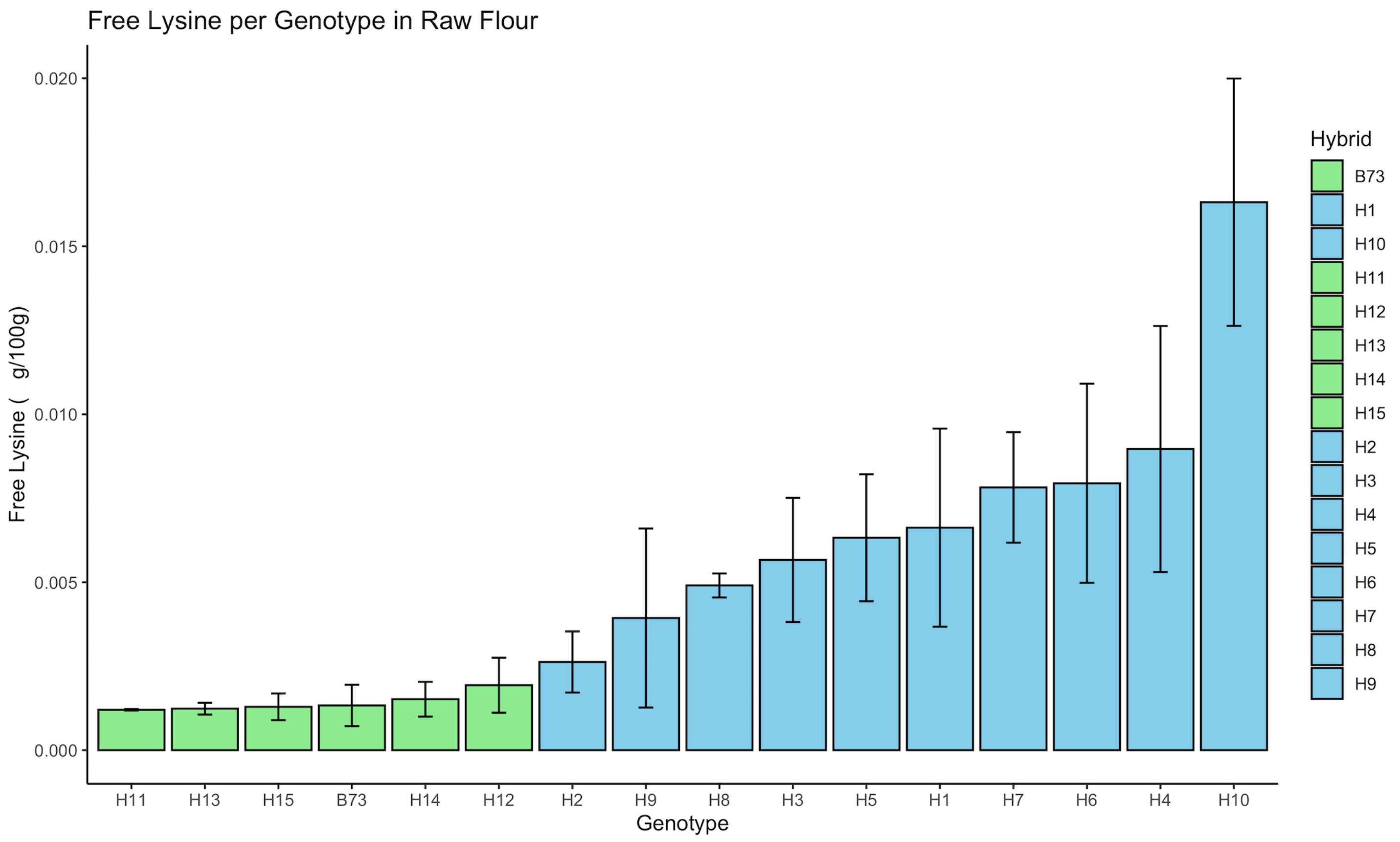

Supplementary Figure 2 | Free lysine content per genotype in raw flour (g/100g). ConAgra derived lines (green columns) had significantly lesser free lysine in raw flour compared to QPP hybrids. Hybrids differed more in free lysine content compared to protein-bound lysine, but g/100g measurements were significantly lesser in free lysine than protein-bound (<3% compared to protein-bound).

Cultivar	Ref. No.	Plant Height		# Ears/Plant		Ear Length	
		cm	*sd*	#	*sd*	cm	*sd*
BC_2F_5 F_1 Hybrid	**H1**	6.92	*1.33*	1.09	*0.27*	20.62	*1.52*
	**H2**	6.94	*1.08*	1.58	*0.36*	21.78	*0.90*
	**H3**	7.28	*1.41*	1.38	*0.32*	22.41	*1.71*
	**H4**	7.08	*1.36*	1.02	*0.07*	22.06	*1.54*
	**H5**	6.94	*1.25*	1.13	*0.22*	19.57	*1.10*
average		***7.03***	***1.24***	***1.24***	***0.33***	***21.29***	***1.70***
BC_3F_4 F_1 Hybrid	**H6**	7.35	*1.05*	1.16	*0.27*	21.03	*0.92*
	**H7**	7.19	*1.29*	1.34	*0.35*	19.92	*1.24*
	**H8**	7.59	*1.26*	1.11	*0.27*	20.82	*1.61*
	**H9**	7.43	*1.30*	1.12	*0.17*	20.73	*1.14*
	**H10**	6.62	*1.14*	1.38	*0.43*	19.39	*0.74*
average		***7.23***	***1.20***	***1.22***	***0.32***	***20.38***	***1.28***
ConAgra® Brands Popcorn	**H11**	7.16	*1.24*	1.49	*0.42*	20.62	*1.33*
	**H12**	6.98	*1.41*	1.70	*0.38*	20.98	*2.26*
	**H13**	6.87	*1.17*	1.38	*0.40*	18.57	*1.18*
	**H14**	6.79	*1.14*	1.78	*0.30*	20.74	*1.22*
	**H15**	6.73	*0.95*	1.42	*0.42*	20.02	*0.90*
average		***6.91***	***1.15***	***1.55***	***0.40***	***20.19***	***1.64***

Supplementary Table 1. Averaged values of hand measured traits from all cultivars and by grouping.

Backcross	Hybrid	Type	Arg	Asx	Glx	Gly	His	Ile	Leu	Lys	Met	Phe	Pro	Ser	Thr	Tyr	Val	Total
BC2	H1	*Raw*	0.520	0.807	1.562	1.062	0.367	0.340	0.872	0.329	0.101	0.400	0.914	0.414	0.443	0.286	0.442	8.859
			0.567	0.947	1.550	0.880	0.372	0.345	0.810	0.376	0.102	0.396	0.848	0.396	0.449	0.300	0.462	8.8
			0.697	1.005	1.876	1.285	0.425	0.435	1.077	0.408	0.126	0.487	1.145	0.553	0.602	0.362	0.555	11.038
		Air	0.382	0.751	1.897	1.150	0.394	0.386	1.114	0.211	0.112	0.448	1.084	0.461	0.516	0.324	0.453	9.683
			0.378	0.703	1.733	0.959	0.354	0.355	0.987	0.217	0.126	0.403	1.012	0.412	0.473	0.278	0.440	8.83
			0.484	0.716	1.688	0.978	0.345	0.338	0.907	0.236	0.113	0.409	0.947	0.408	0.439	0.284	0.443	8.735
	H2	*Raw*	0.329	0.647	1.946	1.036	0.261	0.392	1.232	0.215	0.164	0.442	0.845	0.474	0.475	0.352	0.387	9.197
			0.434	0.801	1.849	1.170	0.345	0.410	1.098	0.279	0.134	0.438	1.037	0.462	0.530	0.346	0.459	9.792
			0.326	0.656	1.661	0.908	0.273	0.404	1.262	0.209	0.172	0.481	0.848	0.474	0.439	0.369	0.380	8.862
		Air	0.327	0.695	1.996	1.351	0.327	0.381	1.203	0.175	0.153	0.442	0.970	0.467	0.448	0.330	0.427	9.692
			0.305	0.654	1.776	0.988	0.332	0.331	1.028	0.176	0.131	0.398	0.959	0.426	0.470	0.298	0.411	8.683
			0.275	0.590	1.661	1.058	0.315	0.347	1.079	0.140	0.118	0.419	0.934	0.373	0.389	0.286	0.389	8.373
	H3	*Raw*	0.397	0.725	1.520	0.958	0.345	0.351	1.046	0.266	0.102	0.433	1.042	0.455	0.461	0.330	0.433	8.864
			0.566	0.876	1.832	1.078	0.360	0.413	1.111	0.332	0.131	0.452	1.063	0.494	0.559	0.337	0.484	10.088
			0.582	0.893	1.722	1.336	0.393	0.355	0.868	0.353	0.129	0.418	0.987	0.448	0.528	0.302	0.479	9.793
		Air	0.473	0.756	1.640	1.093	0.369	0.370	1.031	0.243	0.116	0.421	1.134	0.448	0.495	0.301	0.460	9.35
			0.458	0.750	1.785	1.360	0.390	0.367	1.072	0.234	0.119	0.434	1.204	0.480	0.544	0.311	0.502	10.01
			0.521	0.779	1.931	1.189	0.397	0.407	1.092	0.247	0.122	0.450	1.153	0.515	0.557	0.325	0.523	10.208
	H4	*Raw*	0.668	0.869	1.972	1.197	0.379	0.510	1.283	0.357	0.149	0.523	1.093	0.578	0.602	0.372	0.546	11.098
			0.588	0.732	1.657	1.075	0.349	0.382	0.919	0.344	0.114	0.431	0.900	0.446	0.451	0.306	0.473	9.167
			0.425	0.602	1.157	0.784	0.317	0.298	0.767	0.265	0.093	0.347	0.784	0.370	0.385	0.257	0.385	7.236
		Air	0.611	0.855	2.222	1.185	0.386	0.452	1.157	0.314	0.152	0.495	1.152	0.523	0.586	0.348	0.543	10.981
			0.605	0.886	2.038	1.071	0.376	0.476	1.195	0.323	0.133	0.504	1.157	0.503	0.545	0.372	0.561	10.745
			0.651	0.900	2.277	1.340	0.384	0.518	1.312	0.339	0.149	0.522	1.284	0.570	0.602	0.362	0.579	11.789
	H5	*Raw*	0.469	0.736	1.406	0.878	0.301	0.325	0.827	0.331	0.113	0.409	0.856	0.442	0.481	0.283	0.423	8.28
			0.614	0.964	2.215	1.185	0.382	0.462	1.138	0.384	0.150	0.499	1.204	0.523	0.566	0.365	0.531	11.182
			0.660	1.068	1.838	1.170	0.372	0.469	1.120	0.390	0.175	0.476	1.172	0.516	0.584	0.338	0.562	10.91
		Air	0.578	0.872	1.760	1.138	0.359	0.428	1.077	0.300	0.149	0.461	1.006	0.514	0.539	0.315	0.499	9.995
			0.458	0.747	1.899	1.158	0.351	0.387	1.057	0.231	0.126	0.435	1.042	0.415	0.476	0.305	0.462	9.549
			0.507	0.785	1.706	1.069	0.339	0.382	0.975	0.266	0.132	0.412	0.999	0.431	0.476	0.292	0.468	9.239

Supplementary Table 2. Protein-bound amino acid profiles of Quality Protein Popcorn BC_2F_5-derived hybrids. Three replicates of raw flour and air-popped flakes were submitted for analysis (g/100g).

Backcross	Hybrid	Type	Arg	Asx	Glx	Gly	His	Ile	Leu	Lys	Met	Phe	Pro	Ser	Thr	Tyr	Val	Total
BC3	H6	*Raw*	0.539	0.876	2.074	1.081	0.352	0.433	1.131	0.272	0.131	0.452	1.224	0.456	0.572	0.318	0.514	10.425
			0.643	0.951	1.958	1.174	0.363	0.429	0.995	0.380	0.140	0.438	1.142	0.530	0.594	0.311	0.535	10.583
			0.602	1.042	1.970	1.105	0.363	0.373	0.914	0.359	0.125	0.424	1.033	0.480	0.518	0.293	0.490	10.091
		Air	0.468	0.846	2.166	1.126	0.356	0.396	1.006	0.245	0.125	0.425	1.069	0.429	0.513	0.314	0.485	9.969
			0.529	0.929	2.007	1.139	0.360	0.434	1.100	0.266	0.132	0.465	1.188	0.464	0.559	0.320	0.523	10.415
			0.444	0.838	2.010	1.152	0.340	0.428	1.103	0.238	0.122	0.446	1.167	0.434	0.510	0.314	0.513	10.059
	H7	*Raw*	0.443	0.836	1.531	0.931	0.304	0.306	0.813	0.287	0.126	0.368	0.903	0.406	0.461	0.278	0.397	8.39
			0.474	0.852	1.740	0.936	0.329	0.364	0.893	0.294	0.138	0.396	0.983	0.406	0.480	0.306	0.452	9.043
			0.569	0.882	1.487	1.028	0.324	0.318	0.813	0.321	0.126	0.382	0.895	0.444	0.476	0.265	0.442	8.772
		Air	0.538	0.856	1.919	1.154	0.364	0.405	1.027	0.259	0.136	0.444	1.059	0.462	0.510	0.300	0.488	9.921
			0.397	0.818	1.801	0.939	0.336	0.343	0.911	0.227	0.133	0.385	1.010	0.385	0.470	0.262	0.449	8.866
			0.397	0.714	1.587	0.902	0.321	0.319	0.842	0.204	0.116	0.359	0.971	0.392	0.426	0.235	0.406	8.191
	H8	*Raw*	0.536	0.864	1.544	1.022	0.332	0.342	0.841	0.290	0.132	0.396	1.026	0.433	0.447	0.274	0.446	8.925
			0.645	0.916	1.788	1.234	0.346	0.393	0.907	0.350	0.146	0.441	1.046	0.503	0.499	0.296	0.503	10.013
			0.466	0.821	1.619	0.846	0.329	0.322	0.834	0.245	0.108	0.368	0.930	0.393	0.412	0.263	0.431	8.387
		Air	0.510	0.797	1.940	1.048	0.349	0.357	0.899	0.252	0.117	0.371	1.101	0.401	0.498	0.265	0.478	9.383
			0.370	0.728	1.769	1.054	0.321	0.326	0.849	0.197	0.116	0.359	1.019	0.381	0.438	0.253	0.437	8.617
			0.419	0.615	1.562	0.980	0.339	0.344	0.903	0.200	0.106	0.370	1.075	0.407	0.475	0.247	0.454	8.496
	H9	*Raw*	0.577	0.936	1.861	1.173	0.325	0.391	0.955	0.315	0.140	0.410	1.130	0.475	0.545	0.280	0.491	10.004
			0.524	0.843	1.570	0.929	0.329	0.351	0.878	0.285	0.133	0.396	0.924	0.445	0.452	0.283	0.450	8.792
			0.684	0.907	1.852	1.266	0.328	0.449	1.089	0.375	0.180	0.457	1.141	0.545	0.593	0.328	0.542	10.736
		Air	0.497	0.727	1.969	0.912	0.330	0.374	0.932	0.237	0.125	0.407	1.082	0.423	0.486	0.264	0.478	9.243
			0.435	0.707	1.697	0.950	0.335	0.357	0.938	0.216	0.119	0.394	1.104	0.423	0.460	0.267	0.452	8.854
			0.412	0.605	1.500	0.789	0.324	0.332	0.873	0.193	0.115	0.381	1.000	0.410	0.465	0.259	0.425	8.083
	H10	*Raw*	0.512	0.938	1.740	1.193	0.321	0.364	0.885	0.310	0.154	0.405	1.034	0.435	0.486	0.296	0.459	9.532
			0.485	0.956	1.554	0.878	0.288	0.358	0.810	0.303	0.130	0.397	0.861	0.407	0.462	0.277	0.428	8.594
			0.640	1.064	2.069	1.304	0.326	0.427	1.005	0.376	0.180	0.433	1.135	0.535	0.606	0.323	0.528	10.951
		Air	0.492	0.940	2.046	1.006	0.344	0.392	0.966	0.267	0.165	0.430	1.032	0.431	0.488	0.291	0.480	9.77
			0.370	0.783	1.800	1.115	0.338	0.324	0.835	0.193	0.120	0.379	0.931	0.392	0.423	0.260	0.423	8.686
			0.385	0.768	1.891	1.036	0.310	0.331	0.842	0.208	0.133	0.374	0.969	0.387	0.458	0.257	0.438	8.787

Supplementary Table 3. Protein-bound amino acid profiles of Quality Protein Popcorn BC_3F_4-derived hybrids. Three replicates of raw flour and air-popped flakes were submitted for analysis (g/100g).

Backcross	Hybrid	Type	Arg	Asx	Glx	Gly	His	Ile	Leu	Lys	Met	Phe	Pro	Ser	Thr	Tyr	Val	Total
ConAgra Hybrids	H11	*Raw*	0.320	0.591	1.741	0.963	0.216	0.395	1.265	0.171	0.166	0.460	0.815	0.416	0.385	0.343	0.353	8.6
			0.327	0.644	2.201	0.999	0.248	0.442	1.383	0.186	0.198	0.483	0.959	0.487	0.453	0.400	0.394	9.804
			0.355	0.591	1.756	0.907	0.225	0.359	1.064	0.212	0.160	0.417	0.735	0.395	0.384	0.321	0.352	8.233
		Air	0.318	0.615	2.255	0.986	0.243	0.506	1.580	0.114	0.187	0.508	1.020	0.509	0.441	0.368	0.415	10.065
			0.321	0.640	2.124	1.173	0.253	0.498	1.551	0.119	0.218	0.493	0.986	0.547	0.497	0.373	0.413	10.206
			0.342	0.628	1.999	1.176	0.242	0.455	1.440	0.127	0.172	0.515	0.937	0.510	0.440	0.356	0.386	9.725
	H12	*Raw*	0.347	0.736	2.244	1.173	0.259	0.622	1.859	0.165	0.225	0.605	1.178	0.623	0.565	0.449	0.479	11.529
			0.419	0.712	1.944	1.277	0.269	0.492	1.439	0.229	0.200	0.536	0.921	0.547	0.493	0.365	0.432	10.275
			0.363	0.748	2.411	1.362	0.264	0.604	1.848	0.160	0.245	0.606	1.154	0.645	0.574	0.457	0.456	11.897
		Air	0.348	0.705	2.385	1.332	0.270	0.608	1.795	0.146	0.220	0.583	1.193	0.597	0.527	0.439	0.456	11.604
			0.308	0.680	2.387	1.210	0.265	0.572	1.810	0.113	0.214	0.592	1.168	0.568	0.486	0.430	0.439	11.242
			0.319	0.652	2.592	1.186	0.252	0.563	1.729	0.115	0.211	0.570	1.089	0.550	0.511	0.407	0.435	11.181
	H13	*Raw*	0.379	0.732	2.690	1.294	0.303	0.578	1.763	0.163	0.200	0.580	1.251	0.568	0.564	0.455	0.516	12.036
			0.284	0.601	2.010	1.003	0.245	0.468	1.411	0.171	0.160	0.497	0.938	0.471	0.467	0.388	0.388	9.502
			0.344	0.679	2.417	1.210	0.265	0.616	1.868	0.148	0.207	0.634	1.123	0.561	0.546	0.464	0.456	11.538
		Air	0.333	0.707	2.317	1.198	0.258	0.581	1.756	0.140	0.225	0.582	1.083	0.529	0.485	0.428	0.460	11.082
			0.356	0.752	2.391	1.198	0.262	0.617	1.791	0.135	0.221	0.591	1.153	0.607	0.517	0.426	0.467	11.484
			0.298	0.594	2.051	1.149	0.250	0.488	1.525	0.125	0.195	0.507	0.967	0.497	0.449	0.356	0.401	9.852
	H14	*Raw*	0.318	0.653	2.273	1.143	0.266	0.559	1.714	0.154	0.198	0.576	1.083	0.524	0.475	0.421	0.441	10.798
			0.409	0.724	2.258	1.230	0.271	0.523	1.619	0.176	0.187	0.541	1.050	0.545	0.475	0.386	0.441	10.835
			0.484	0.821	1.954	1.170	0.269	0.537	1.480	0.261	0.211	0.546	1.033	0.579	0.537	0.399	0.467	10.748
		Air	0.303	0.618	2.206	0.974	0.244	0.479	1.539	0.107	0.193	0.538	0.982	0.531	0.441	0.356	0.394	9.905
			0.361	0.742	2.174	1.172	0.278	0.573	1.732	0.131	0.192	0.582	1.140	0.553	0.480	0.405	0.465	10.98
			0.345	0.712	2.305	1.138	0.272	0.541	1.713	0.141	0.211	0.560	1.080	0.594	0.498	0.420	0.457	10.987
	H15	*Raw*	0.343	0.670	2.050	1.074	0.256	0.474	1.458	0.187	0.190	0.509	0.997	0.553	0.500	0.395	0.423	10.079
			0.328	0.653	1.900	0.990	0.249	0.429	1.315	0.210	0.182	0.476	0.943	0.465	0.475	0.371	0.382	9.368
			0.409	0.691	1.620	0.947	0.243	0.410	1.169	0.244	0.156	0.456	0.810	0.444	0.403	0.331	0.378	8.711
		Air	0.331	0.534	1.612	0.860	0.241	0.370	1.193	0.126	0.172	0.447	0.841	0.405	0.363	0.302	0.350	8.147
			0.269	0.559	1.833	1.043	0.232	0.398	1.269	0.113	0.161	0.450	0.835	0.454	0.386	0.308	0.355	8.665
			0.372	0.667	1.899	1.087	0.252	0.440	1.280	0.163	0.189	0.470	0.889	0.491	0.442	0.331	0.404	9.376
B73 Reference			0.535	0.779	2.204	1.262	0.276	0.544	1.498	0.268	0.209	0.543	1.029	0.606	0.526	0.390	0.492	11.161
			0.575	0.838	2.266	1.193	0.290	0.628	1.731	0.257	0.213	0.599	1.171	0.595	0.530	0.418	0.515	11.819
			0.203	0.813	2.107	1.228	0.290	0.521	1.425	0.316	0.192	0.540	0.995	0.577	0.508	0.402	0.492	10.609

Supplementary Table 4. Protein-bound amino acid profiles of ConAgra derived hybrids and B73 for reference. Three replicates of raw flour and air-popped flakes were submitted for analysis (g/100g).

	Arg	*sd*	Asx	*sd*	Glx	*sd*	Gly	*sd*	His	*sd*	Ile	*sd*	Leu	*sd*	Lys	*sd*	Met	*sd*	Phe	*sd*	Pro	*sd*	Ser	*sd*	Thr	*sd*	Tyr	*sd*	Val	*sd*
**H1**	0.595	*0.091*	0.920	*0.102*	1.662	*0.185*	1.075	*0.203*	0.388	*0.032*	0.373	*0.054*	0.920	*0.140*	0.371 *a*	*0.039*	0.110	*0.014*	0.428	*0.051*	0.969	*0.156*	0.455	*0.086*	0.498	*0.090*	0.316	*0.041*	0.486	*0.060*
**H2**	0.363	*0.061*	0.701	*0.086*	1.819	*0.145*	1.038	*0.131*	0.293	*0.046*	0.402	*0.009*	1.198	*0.087*	0.234 *bcdef*	*0.039*	0.157	*0.020*	0.454	*0.024*	0.910	*0.110*	0.470	*0.007*	0.481	*0.046*	0.355	*0.012*	0.409	*0.044*
**H3**	0.515	*0.103*	0.831	*0.093*	1.691	*0.158*	1.124	*0.193*	0.366	*0.025*	0.373	*0.035*	1.008	*0.125*	0.317 *abcd*	*0.045*	0.121	*0.016*	0.434	*0.017*	1.031	*0.039*	0.465	*0.025*	0.516	*0.050*	0.323	*0.018*	0.465	*0.028*
**H4**	0.560	*0.124*	0.734	*0.134*	1.595	*0.411*	1.019	*0.212*	0.348	*0.031*	0.397	*0.107*	0.989	*0.265*	0.322 *abc*	*0.050*	0.119	*0.028*	0.434	*0.088*	0.926	*0.156*	0.465	*0.105*	0.479	*0.111*	0.312	*0.058*	0.468	*0.081*
**H5**	0.581	*0.099*	0.923	*0.170*	1.820	*0.405*	1.077	*0.173*	0.352	*0.044*	0.419	*0.081*	1.028	*0.174*	0.368 *a*	*0.033*	0.146	*0.031*	0.461	*0.047*	1.077	*0.193*	0.494	*0.045*	0.544	*0.055*	0.329	*0.042*	0.505	*0.073*
**H6**	0.595	*0.052*	0.956	*0.083*	2.001	*0.064*	1.120	*0.048*	0.360	*0.006*	0.412	*0.034*	1.013	*0.109*	0.337 *ab*	*0.057*	0.132	*0.007*	0.438	*0.014*	1.133	*0.096*	0.489	*0.038*	0.561	*0.039*	0.307	*0.013*	0.513	*0.023*
**H7**	0.495	*0.066*	0.856	*0.023*	1.586	*0.135*	0.965	*0.055*	0.319	*0.013*	0.329	*0.031*	0.840	*0.046*	0.301 *abcde*	*0.018*	0.130	*0.007*	0.382	*0.014*	0.927	*0.049*	0.419	*0.022*	0.472	*0.010*	0.283	*0.021*	0.431	*0.029*
**H8**	0.549	*0.090*	0.867	*0.048*	1.650	*0.125*	1.034	*0.195*	0.336	*0.009*	0.352	*0.037*	0.861	*0.040*	0.295 *abcde*	*0.053*	0.129	*0.019*	0.402	*0.036*	1.001	*0.062*	0.443	*0.056*	0.453	*0.044*	0.277	*0.017*	0.460	*0.038*
**H9**	0.595	*0.081*	0.896	*0.048*	1.761	*0.166*	1.123	*0.174*	0.327	*0.002*	0.397	*0.049*	0.974	*0.107*	0.325 *abc*	*0.046*	0.151	*0.025*	0.421	*0.032*	1.065	*0.122*	0.488	*0.051*	0.530	*0.072*	0.297	*0.027*	0.495	*0.046*
**H10**	0.546	*0.083*	0.986	*0.068*	1.788	*0.261*	1.125	*0.221*	0.312	*0.020*	0.383	*0.038*	0.900	*0.098*	0.330 *abc*	*0.040*	0.155	*0.025*	0.412	*0.019*	1.010	*0.139*	0.459	*0.067*	0.518	*0.077*	0.299	*0.023*	0.472	*0.051*
**H11**	0.334	*0.019*	0.609	*0.030*	1.899	*0.261*	0.956	*0.046*	0.230	*0.017*	0.399	*0.041*	1.237	*0.161*	0.190 *ef*	*0.021*	0.175	*0.020*	0.453	*0.034*	0.836	*0.113*	0.433	*0.048*	0.407	*0.040*	0.355	*0.041*	0.366	*0.024*
**H12**	0.376	*0.038*	0.732	*0.018*	2.200	*0.236*	1.271	*0.095*	0.264	*0.005*	0.573	*0.071*	1.715	*0.239*	0.185 *ef*	*0.038*	0.224	*0.023*	0.583	*0.040*	1.084	*0.142*	0.605	*0.051*	0.544	*0.044*	0.424	*0.051*	0.456	*0.024*
**H13**	0.336	*0.048*	0.671	*0.066*	2.372	*0.342*	1.169	*0.150*	0.271	*0.029*	0.554	*0.077*	1.681	*0.240*	0.161 *f*	*0.012*	0.189	*0.025*	0.570	*0.069*	1.104	*0.157*	0.533	*0.054*	0.526	*0.052*	0.435	*0.042*	0.453	*0.064*
**H14**	0.404	*0.083*	0.733	*0.084*	2.162	*0.180*	1.181	*0.045*	0.269	*0.002*	0.540	*0.018*	1.604	*0.118*	0.197 *def*	*0.057*	0.199	*0.012*	0.554	*0.019*	1.055	*0.026*	0.549	*0.028*	0.496	*0.036*	0.402	*0.018*	0.450	*0.015*
**H15**	0.360	*0.043*	0.671	*0.019*	1.857	*0.218*	1.004	*0.064*	0.249	*0.007*	0.438	*0.033*	1.314	*0.144*	0.214 *cdef*	*0.029*	0.176	*0.018*	0.480	*0.027*	0.917	*0.096*	0.487	*0.058*	0.459	*0.050*	0.366	*0.033*	0.395	*0.025*
**B73**	0.438	*0.204*	0.810	*0.030*	2.192	*0.080*	1.228	*0.034*	0.285	*0.008*	0.564	*0.056*	1.552	*0.160*	0.280 *abcdef*	*0.031*	0.205	*0.011*	0.561	*0.033*	1.065	*0.093*	0.593	*0.015*	0.521	*0.012*	0.404	*0.014*	0.500	*0.013*

Supplementary Table 5. Averaged protein-bound amino acid values from ground flour from all tested cultivars (g/100g total weight).

Protein-Bound Lysine Averages in Ground Flour and Air-Popped Flakes

	**Ground Flour**	***sd***	**Air-Popped Flakes**	***sd***
**H1**	0.371[a]	*0.039*	0.221[bc]	*0.013*
**H2**	0.234[bcdef]	*0.039*	0.164[cd]	*0.021*
**H3**	0.317[abcd]	*0.045*	0.241[b]	*0.007*
**H4**	0.322[abc]	*0.050*	0.325[a]	*0.012*
**H5**	0.368[a]	*0.033*	0.266[ab]	*0.035*
average	**0.323[a]**	***0.055***	**0.243[a]**	***0.059***
**H6**	0.337[ab]	*0.057*	0.250[b]	*0.014*
**H7**	0.301[abcde]	*0.018*	0.230[bc]	*0.027*
**H8**	0.295[abcde]	*0.053*	0.216[bc]	*0.031*
**H9**	0.325[abc]	*0.046*	0.215[bc]	*0.022*
**H10**	0.330[abc]	*0.040*	0.223[bc]	*0.039*
average	**0.317[a]**	***0.019***	**0.227[a]**	***0.014***
**H11**	0.190[ef]	*0.021*	0.120[d]	*0.006*
**H12**	0.185[ef]	*0.038*	0.125[d]	*0.019*
**H13**	0.161[f]	*0.012*	0.133[d]	*0.007*
**H14**	0.197[def]	*0.057*	0.126[d]	*0.017*
**H15**	0.214[cdef]	*0.029*	0.134[d]	*0.026*
average	**0.189[b]**	***0.019***	**0.128[b]**	**0.006**
**B73**	0.280[abcdef]	*0.031*	*NA*	*NA*
	**Pearson's Correlation**	**0.872**	*t = 6.42*<br>*df = 13*<br>*p-vale = 0.0000227*	

Supplementary Table 6. Protein-bound lysine averages of all popcorn cultivars and B73 in ground flour and air-popped flake samples (g/100g). Significance between cultivar measurements and by group is indicated by lettered superscripts. Pearson's correlation test between lysine measurements in ground flour and air-popped flakes is also shown.

	Hybrid	Type	Ala	Arg	Asn	Asp	Gln	Glu	Gly	His	Ile	Leu	Lys	Met	Phe	Pro	Ser	Trp	Thr	Tyr	Val	Cys	Total
BC2	H1	Raw	0.0114	0.0148	0.1276	0.0810	0.0205	0.1123	0.0708	0.0019	0.0010	0.0012	0.0038	0.0006	0.0021	0.1458	0.0059	0.0037	0.0234	0.0128	0.0047	0.0011	0.6464
			0.0091	0.0364	0.3190	0.0831	0.0325	0.1237	0.0723	0.0030	0.0010	0.0012	0.0064	0.0004	0.0024	0.1846	0.0064	0.0046	0.0302	0.0162	0.0048	0.0014	0.9387
			0.0103	0.0488	0.3212	0.0758	0.0108	0.0794	0.0764	0.0033	0.0008	0.0009	0.0097	0.0002	0.0014	0.2608	0.0033	0.0022	0.0360	0.0102	0.0050	0.0017	0.9582
		Air	0.0170	0.0126	0.1589	0.0849	0.0020	0.0918	0.0709	0.0015	0.0030	0.0018	0.0041	0.0010	0.0026	0.0889	0.0077	0.0036	0.0206	0.0137	0.0062	0.0008	0.5936
			0.0129	0.0100	0.1002	0.1029	0.0032	0.0889	0.0677	0.0016	0.0029	0.0021	0.0049	0.0014	0.0030	0.0778	0.0081	0.0042	0.0173	0.0166	0.0072	0.0006	0.5335
			0.0100	0.0108	0.0873	0.0828	0.0002	0.0661	0.0632	0.0014	0.0018	0.0017	0.0032	0.0011	0.0023	0.1123	0.0067	0.0043	0.0184	0.0150	0.0054	0.0006	0.4946
	H2	Raw	0.0115	0.0120	0.0422	0.0281	0.0023	0.0242	0.0471	0.0010	0.0006	0.0007	0.0030	0.0004	0.0008	0.1230	0.0048	0.0102	0.0176	0.0063	0.0027	0.0009	0.3394
			0.0094	0.0090	0.1426	0.1061	0.0450	0.1522	0.0742	0.0015	0.0008	0.0019	0.0033	0.0008	0.0024	0.0982	0.0067	0.0032	0.0192	0.0167	0.0045	0.0009	0.6986
			0.0111	0.0054	0.0372	0.0320	0.0080	0.0328	0.0347	0.0009	0.0004	0.0009	0.0016	0.0006	0.0008	0.0654	0.0056	0.0028	0.0102	0.0049	0.0029	0.0005	0.2587
		Air	0.0153	0.0063	0.0721	0.0886	0.0060	0.0717	0.0576	0.0012	0.0024	0.0033	0.0040	0.0008	0.0029	0.0635	0.0087	0.0078	0.0130	0.0155	0.0068	0.0005	0.448
			0.0099	0.0058	0.0587	0.1114	0.0056	0.0945	0.0642	0.0014	0.0016	0.0026	0.0044	0.0010	0.0034	0.0601	0.0073	0.0040	0.0131	0.0206	0.0060	0.0006	0.4762
			0.0129	0.0043	0.0542	0.0939	0.0052	0.0843	0.0614	0.0012	0.0024	0.0033	0.0041	0.0010	0.0031	0.0503	0.0085	0.0043	0.0123	0.0163	0.0074	0.0005	0.4309
	H3	Raw	0.0058	0.0134	0.1201	0.0865	0.0147	0.0830	0.0705	0.0019	0.0006	0.0009	0.0038	0.0002	0.0016	0.1229	0.0025	0.0030	0.0196	0.0096	0.0028	0.0009	0.5643
			0.0065	0.0239	0.2047	0.0855	0.0251	0.1017	0.0662	0.0020	0.0008	0.0012	0.0058	0.0004	0.0021	0.1495	0.0048	0.0020	0.0228	0.0124	0.0040	0.0011	0.7225
			0.0114	0.0265	0.1967	0.0993	0.0316	0.1397	0.0726	0.0027	0.0026	0.0020	0.0074	0.0011	0.0027	0.1650	0.0080	0.0065	0.0309	0.0142	0.0072	0.0015	0.8296
		Air	0.0081	0.0174	0.1245	0.1006	0.0038	0.1078	0.0736	0.0019	0.0011	0.0016	0.0043	0.0008	0.0027	0.1258	0.0060	0.0040	0.0216	0.0149	0.0050	0.0010	0.6265
			0.0085	0.0140	0.1158	0.1098	0.0011	0.0856	0.0647	0.0020	0.0010	0.0014	0.0045	0.0006	0.0032	0.1328	0.0049	0.0043	0.0211	0.0156	0.0041	0.0009	0.5959
			0.0075	0.0137	0.1087	0.0868	0.0008	0.0674	0.0676	0.0016	0.0011	0.0014	0.0037	0.0008	0.0018	0.1205	0.0042	0.0019	0.0199	0.0100	0.0047	0.0008	0.5249
	H4	Raw	0.0107	0.0225	0.0641	0.0177	0.0041	0.0182	0.0511	0.0027	0.0025	0.0055	0.0131	0.0024	0.0020	0.1237	0.0071	0.0011	0.0195	0.0093	0.0063	0.0008	0.3844
			0.0046	0.0119	0.0497	0.0353	0.0023	0.0312	0.0360	0.0017	0.0008	0.0023	0.0060	0.0010	0.0008	0.0669	0.0032	0.0010	0.0101	0.0055	0.0027	0.0005	0.2735
			0.0065	0.0181	0.0636	0.0303	0.0028	0.0466	0.0509	0.0021	0.0012	0.0024	0.0078	0.0011	0.0010	0.0755	0.0045	0.0012	0.0139	0.0066	0.0033	0.0006	0.34
		Air	0.0030	0.0085	0.0690	0.0526	0.0003	0.0561	0.0588	0.0010	0.0002	0.0005	0.0029	0.0004	0.0010	0.0912	0.0022	0.0013	0.0136	0.0072	0.0022	0.0005	0.3725
			0.0049	0.0119	0.1013	0.0653	0.0005	0.0613	0.0688	0.0015	0.0000	0.0003	0.0035	0.0002	0.0008	0.1670	0.0026	0.0014	0.0226	0.0062	0.0021	0.0009	0.5231
			0.0040	0.0092	0.1275	0.0474	0.0002	0.0500	0.0548	0.0011	0.0000	0.0004	0.0032	0.0002	0.0004	0.1270	0.0025	0.0009	0.0182	0.0044	0.0017	0.0006	0.4537
	H5	Raw	0.0146	0.0242	0.2335	0.1131	0.0175	0.1100	0.0755	0.0029	0.0010	0.0012	0.0061	0.0004	0.0025	0.2282	0.0039	0.0048	0.0349	0.0186	0.0054	0.0018	0.9001
			0.0145	0.0104	0.1155	0.0842	0.0113	0.1040	0.0743	0.0021	0.0019	0.0014	0.0045	0.0004	0.0018	0.1644	0.0046	0.0024	0.0239	0.0157	0.0050	0.0014	0.6437
			0.0174	0.0256	0.3523	0.1178	0.0157	0.1078	0.0790	0.0033	0.0017	0.0020	0.0083	0.0006	0.0027	0.2296	0.0055	0.0023	0.0360	0.0166	0.0062	0.0018	1.0322
		Air	0.0185	0.0216	0.1768	0.1034	0.0028	0.1032	0.0765	0.0027	0.0011	0.0014	0.0064	0.0004	0.0020	0.2319	0.0049	0.0017	0.0334	0.0142	0.0054	0.0013	0.8096
			0.0130	0.0102	0.0740	0.0913	0.0014	0.0914	0.0682	0.0024	0.0012	0.0014	0.0049	0.0006	0.0020	0.1372	0.0058	0.0038	0.0216	0.0154	0.0046	0.0008	0.5512
			0.0113	0.0211	0.0815	0.0969	0.0015	0.1029	0.0727	0.0028	0.0006	0.0009	0.0055	0.0002	0.0020	0.1537	0.0034	0.0036	0.0254	0.0144	0.0039	0.0010	0.6053

Supplementary Table 7. Free amino acid profiles of Quality Protein Popcorn BC_2F_5-derived hybrids. Three replicates of raw flour and air-popped flakes were submitted for analysis (g/100g).

	Hybrid	Type	Ala	Arg	Asn	Asp	Gln	Glu	Gly	His	Ile	Leu	Lys	Met	Phe	Pro	Ser	Trp	Thr	Tyr	Val	Cys	Total
BC3	H6	Raw	0.0112	0.0117	0.1350	0.1041	0.0459	0.1434	0.0782	0.0021	0.0021	0.0025	0.0053	0.0014	0.0022	0.1157	0.0088	0.0016	0.0239	0.0118	0.0066	0.0013	0.7148
			0.0122	0.0224	0.2299	0.1080	0.0747	0.2250	0.0770	0.0030	0.0022	0.0026	0.0074	0.0021	0.0023	0.1778	0.0101	0.0022	0.0363	0.0171	0.0083	0.0017	1.0223
			0.0168	0.0364	0.3369	0.1340	0.0971	0.2402	0.0778	0.0030	0.0032	0.0038	0.0111	0.0023	0.0043	0.1689	0.0150	0.0022	0.0434	0.0198	0.0111	0.0021	1.2294
		Air	0.0219	0.0137	0.1694	0.1441	0.0026	0.1147	0.0766	0.0025	0.0043	0.0035	0.0073	0.0021	0.0036	0.1542	0.0146	0.0017	0.0313	0.0150	0.0111	0.0012	0.7954
			0.0126	0.0083	0.1368	0.1285	0.0010	0.1011	0.0750	0.0021	0.0021	0.0026	0.0052	0.0012	0.0036	0.0946	0.0072	0.0021	0.0202	0.0146	0.0065	0.0008	0.6261
			0.0064	0.0065	0.1133	0.1263	0.0028	0.1027	0.0772	0.0021	0.0004	0.0014	0.0039	0.0004	0.0028	0.0835	0.0045	0.0018	0.0164	0.0140	0.0029	0.0008	0.5701
	H7	Raw	0.0164	0.0154	0.2389	0.1369	0.0472	0.1368	0.0743	0.0023	0.0027	0.0032	0.0060	0.0012	0.0032	0.0866	0.0082	0.0037	0.0190	0.0172	0.0082	0.0011	0.8285
			0.0189	0.0216	0.2117	0.1363	0.0478	0.1617	0.0765	0.0025	0.0048	0.0042	0.0091	0.0016	0.0042	0.1408	0.0115	0.0028	0.0272	0.0166	0.0114	0.0014	0.9126
			0.0223	0.0269	0.2465	0.1527	0.0752	0.1919	0.0798	0.0029	0.0064	0.0049	0.0084	0.0021	0.0042	0.1546	0.0172	0.0025	0.0325	0.0198	0.0140	0.0016	1.0664
		Air	0.0147	0.0106	0.1190	0.1411	0.0050	0.1054	0.0729	0.0017	0.0047	0.0042	0.0052	0.0014	0.0034	0.0663	0.0087	0.0036	0.0158	0.0144	0.0089	0.0008	0.6078
			0.0373	0.0084	0.1765	0.1483	0.0237	0.1598	0.0776	0.0022	0.0108	0.0081	0.0121	0.0033	0.0060	0.0703	0.0191	0.0055	0.0257	0.0213	0.0179	0.0013	0.8352
			0.0241	0.0106	0.1183	0.1680	0.0122	0.1207	0.0788	0.0023	0.0083	0.0067	0.0094	0.0022	0.0049	0.0823	0.0151	0.0054	0.0215	0.0189	0.0135	0.0010	0.7242
	H8	Raw	0.0072	0.0180	0.2155	0.0931	0.0190	0.0956	0.0703	0.0020	0.0014	0.0010	0.0053	0.0004	0.0023	0.0756	0.0051	0.0037	0.0159	0.0115	0.0038	0.0008	0.6475
			0.0085	0.0180	0.2147	0.0818	0.0151	0.0721	0.0622	0.0020	0.0010	0.0009	0.0047	0.0004	0.0012	0.0812	0.0043	0.0026	0.0145	0.0084	0.0036	0.0008	0.598
			0.0093	0.0161	0.2247	0.1100	0.0359	0.1240	0.0722	0.0017	0.0024	0.0018	0.0047	0.0010	0.0023	0.0624	0.0071	0.0049	0.0154	0.0135	0.0052	0.0008	0.7154
		Air	0.0107	0.0101	0.1340	0.1051	0.0034	0.0841	0.0709	0.0016	0.0026	0.0020	0.0046	0.0009	0.0035	0.0970	0.0062	0.0035	0.0187	0.0140	0.0056	0.0008	0.5793
			0.0200	0.0093	0.1540	0.1419	0.0070	0.1225	0.0742	0.0022	0.0073	0.0048	0.0070	0.0027	0.0045	0.0813	0.0132	0.0041	0.0222	0.0165	0.0125	0.0011	0.7083
			0.0060	0.0053	0.0662	0.0976	0.0003	0.0684	0.0558	0.0015	0.0009	0.0009	0.0024	0.0004	0.0021	0.0828	0.0027	0.0022	0.0157	0.0124	0.0028	0.0005	0.4269
	H9	Raw	0.0091	0.0178	0.2495	0.1091	0.0401	0.1315	0.0740	0.0024	0.0010	0.0014	0.0059	0.0008	0.0018	0.1044	0.0043	0.0040	0.0207	0.0097	0.0043	0.0011	0.7929
			0.0070	0.0017	0.0142	0.0148	0.0012	0.0172	0.0169	0.0005	0.0004	0.0007	0.0009	0.0002	0.0004	0.0366	0.0025	0.0006	0.0051	0.0022	0.0020	0.0002	0.1253
			0.0051	0.0153	0.1639	0.0578	0.0067	0.0579	0.0633	0.0018	0.0006	0.0014	0.0050	0.0008	0.0010	0.1090	0.0044	0.0017	0.0169	0.0078	0.0029	0.0008	0.5241
		Air	0.0130	0.0090	0.1019	0.0989	0.0016	0.0846	0.0718	0.0015	0.0008	0.0016	0.0043	0.0010	0.0018	0.1297	0.0056	0.0014	0.0202	0.0102	0.0044	0.0008	0.5641
			0.0189	0.0110	0.1036	0.1077	0.0021	0.0768	0.0682	0.0018	0.0025	0.0026	0.0066	0.0019	0.0020	0.0862	0.0082	0.0019	0.0168	0.0097	0.0067	0.0006	0.5358
			0.0061	0.0085	0.0711	0.0884	0.0003	0.0517	0.0522	0.0014	0.0002	0.0007	0.0029	0.0002	0.0014	0.0789	0.0017	0.0029	0.0118	0.0081	0.0019	0.0005	0.3909
	H10	Raw	0.0347	0.0328	0.2910	0.1363	0.0989	0.2013	0.0793	0.0034	0.0067	0.0055	0.0202	0.0017	0.0053	0.1161	0.0168	0.0068	0.0317	0.0306	0.0157	0.0018	1.1366
			0.0242	0.0430	0.3838	0.1203	0.1093	0.2285	0.0813	0.0034	0.0074	0.0048	0.0129	0.0018	0.0051	0.1427	0.0160	0.0046	0.0365	0.0273	0.0156	0.0021	1.2706
			0.0434	0.0431	0.2422	0.1473	0.1299	0.2398	0.0796	0.0046	0.0061	0.0046	0.0159	0.0018	0.0057	0.1802	0.0184	0.0040	0.0398	0.0313	0.0153	0.0021	1.2551
		Air	0.0185	0.0189	0.1876	0.1441	0.0024	0.1070	0.0738	0.0024	0.0010	0.0021	0.0069	0.0008	0.0039	0.0898	0.0082	0.0083	0.0183	0.0246	0.0053	0.0008	0.7247
			0.0275	0.0067	0.1387	0.1419	0.0061	0.1155	0.0771	0.0022	0.0060	0.0049	0.0069	0.0025	0.0051	0.0789	0.0187	0.0057	0.0227	0.0321	0.0122	0.0011	0.7125
			0.0260	0.0105	0.1294	0.1455	0.0065	0.1187	0.0765	0.0026	0.0054	0.0044	0.0074	0.0014	0.0048	0.0727	0.0154	0.0084	0.0214	0.0352	0.0126	0.0009	0.7057

Supplementary Table 8. Free amino acid profiles of Quality Protein Popcorn BC_3F_4-derived hybrids. Three replicates of raw flour and air-popped flakes were submitted for analysis (g/100g).

	Hybrid	Type	Ala	Arg	Asn	Asp	Gln	Glu	Gly	His	Ile	Leu	Lys	Met	Phe	Pro	Ser	Trp	Thr	Tyr	Val	Cys	Total
ConAgra Hybrids	H11	Raw	0.0070	0.0026	0.0238	0.0212	0.0015	0.0236	0.0182	0.0007	0.0006	0.0009	0.0012	0.0004	0.0008	0.0342	0.0028	0.0025	0.0057	0.0041	0.0024	0.0003	0.1545
			0.0115	0.0027	0.0243	0.0242	0.0020	0.0279	0.0322	0.0008	0.0008	0.0012	0.0012	0.0006	0.0008	0.0583	0.0044	0.0009	0.0104	0.0039	0.0030	0.0005	0.2116
			0.0062	0.0032	0.0209	0.0166	0.0009	0.0218	0.0251	0.0008	0.0004	0.0007	0.0012	0.0002	0.0006	0.0491	0.0024	0.0016	0.0077	0.0040	0.0019	0.0003	0.1656
		Air	0.0089	0.0016	0.0108	0.0212	0.0003	0.0253	0.0153	0.0006	0.0004	0.0008	0.0012	0.0006	0.0008	0.0257	0.0036	0.0018	0.0049	0.0037	0.0026	0.0002	0.1303
			0.0063	0.0019	0.0113	0.0125	0.0000	0.0126	0.0127	0.0005	0.0002	0.0005	0.0009	0.0002	0.0004	0.0333	0.0019	0.0016	0.0049	0.0025	0.0015	0.0002	0.1059
			0.0054	0.0023	0.0120	0.0201	0.0000	0.0159	0.0209	0.0005	0.0004	0.0007	0.0011	0.0002	0.0006	0.0432	0.0020	0.0014	0.0059	0.0027	0.0019	0.0002	0.1374
	H12	Raw	0.0039	0.0060	0.0202	0.0138	0.0018	0.0207	0.0179	0.0009	0.0004	0.0007	0.0016	0.0004	0.0006	0.0430	0.0018	0.0046	0.0060	0.0038	0.0019	0.0003	0.1503
			0.0048	0.0083	0.0220	0.0093	0.0008	0.0129	0.0182	0.0006	0.0002	0.0004	0.0029	0.0002	0.0004	0.0472	0.0022	0.0059	0.0068	0.0036	0.0015	0.0003	0.1485
			0.0069	0.0045	0.0176	0.0140	0.0014	0.0153	0.0174	0.0006	0.0002	0.0005	0.0014	0.0002	0.0004	0.0473	0.0031	0.0049	0.0068	0.0034	0.0016	0.0003	0.1478
		Air	0.0029	0.0027	0.0070	0.0101	0.0002	0.0110	0.0075	0.0004	0.0002	0.0003	0.0008	0.0002	0.0004	0.0215	0.0012	0.0022	0.0025	0.0026	0.0012	0.0002	0.0751
			0.0039	0.0017	0.0049	0.0151	0.0002	0.0138	0.0044	0.0004	0.0002	0.0005	0.0007	0.0004	0.0004	0.0134	0.0024	0.0022	0.0018	0.0023	0.0012	0.0002	0.0701
			0.0033	0.0014	0.0035	0.0100	0.0000	0.0086	0.0052	0.0003	0.0000	0.0003	0.0007	0.0002	0.0004	0.0131	0.0015	0.0018	0.0016	0.0024	0.0010	0.0002	0.0555
	H13	Raw	0.0037	0.0029	0.0141	0.0175	0.0017	0.0196	0.0140	0.0007	0.0002	0.0005	0.0013	0.0002	0.0006	0.0304	0.0017	0.0045	0.0038	0.0034	0.0015	0.0003	0.1226
			0.0034	0.0035	0.0130	0.0071	0.0013	0.0159	0.0109	0.0005	0.0002	0.0003	0.0010	0.0002	0.0004	0.0222	0.0015	0.0015	0.0027	0.0019	0.0012	0.0002	0.0889
			0.0049	0.0039	0.0132	0.0101	0.0011	0.0168	0.0119	0.0005	0.0002	0.0003	0.0014	0.0004	0.0004	0.0303	0.0023	0.0049	0.0036	0.0033	0.0012	0.0003	0.111
		Air	0.0053	0.0022	0.0073	0.0113	0.0000	0.0108	0.0100	0.0004	0.0002	0.0003	0.0008	0.0002	0.0004	0.0347	0.0021	0.0027	0.0041	0.0026	0.0014	0.0002	0.097
			0.0043	0.0019	0.0054	0.0138	0.0002	0.0135	0.0097	0.0004	0.0002	0.0005	0.0009	0.0004	0.0006	0.0217	0.0023	0.0030	0.0027	0.0025	0.0015	0.0002	0.0857
			0.0048	0.0018	0.0082	0.0118	0.0002	0.0101	0.0087	0.0004	0.0000	0.0003	0.0007	0.0002	0.0004	0.0247	0.0021	0.0011	0.0030	0.0024	0.0012	0.0002	0.0823
	H14	Raw	0.0029	0.0036	0.0274	0.0144	0.0018	0.0211	0.0122	0.0006	0.0004	0.0005	0.0011	0.0002	0.0004	0.0217	0.0014	0.0019	0.0037	0.0025	0.0015	0.0002	0.1195
			0.0035	0.0037	0.0373	0.0259	0.0021	0.0253	0.0184	0.0010	0.0006	0.0007	0.0014	0.0004	0.0008	0.0313	0.0018	0.0029	0.0052	0.0035	0.0022	0.0003	0.1683
			0.0041	0.0081	0.0761	0.0229	0.0021	0.0242	0.0254	0.0015	0.0006	0.0005	0.0021	0.0002	0.0008	0.0532	0.0014	0.0052	0.0079	0.0041	0.0022	0.0005	0.2431
		Air	0.0024	0.0026	0.0175	0.0173	0.0000	0.0153	0.0084	0.0005	0.0002	0.0005	0.0009	0.0002	0.0006	0.0172	0.0009	0.0033	0.0023	0.0025	0.0015	0.0002	0.0943
			0.0028	0.0021	0.0149	0.0175	0.0000	0.0126	0.0083	0.0004	0.0002	0.0005	0.0009	0.0002	0.0004	0.0249	0.0011	0.0026	0.0034	0.0028	0.0015	0.0002	0.0973
			0.0031	0.0021	0.0160	0.0172	0.0000	0.0121	0.0103	0.0005	0.0004	0.0005	0.0010	0.0002	0.0006	0.0272	0.0014	0.0022	0.0036	0.0030	0.0017	0.0002	0.1033
	H15	Raw	0.0060	0.0020	0.0297	0.0196	0.0014	0.0219	0.0158	0.0008	0.0006	0.0009	0.0009	0.0004	0.0006	0.0267	0.0030	0.0012	0.0051	0.0035	0.0019	0.0003	0.1423
			0.0062	0.0025	0.0437	0.0317	0.0022	0.0282	0.0249	0.0009	0.0012	0.0012	0.0014	0.0006	0.0008	0.0301	0.0033	0.0018	0.0068	0.0049	0.0031	0.0003	0.1958
			0.0085	0.0043	0.0499	0.0400	0.0038	0.0282	0.0365	0.0009	0.0013	0.0013	0.0016	0.0008	0.0010	0.0524	0.0051	0.0012	0.0103	0.0054	0.0035	0.0005	0.2565
		Air	0.0039	0.0018	0.0142	0.0331	0.0002	0.0208	0.0093	0.0007	0.0010	0.0009	0.0010	0.0006	0.0008	0.0255	0.0025	0.0015	0.0043	0.0042	0.0021	0.0002	0.1286
			0.0067	0.0026	0.0197	0.0333	0.0005	0.0227	0.0189	0.0007	0.0015	0.0012	0.0012	0.0006	0.0008	0.0369	0.0036	0.0021	0.0067	0.0049	0.0032	0.0002	0.168
			0.0076	0.0028	0.0358	0.0396	0.0003	0.0286	0.0232	0.0009	0.0019	0.0016	0.0014	0.0006	0.0010	0.0355	0.0043	0.0014	0.0078	0.0060	0.0041	0.0003	0.2047
B73 Reference			0.0086	0.0031	0.0137	0.0076	0.0002	0.0070	0.0249	0.0007	0.0002	0.0004	0.0011	0.0000	0.0006	0.0580	0.0014	0.0003	0.0066	0.0022	0.0017	0.0005	0.1388
			0.0092	0.0026	0.0129	0.0103	0.0002	0.0098	0.0267	0.0007	0.0002	0.0004	0.0009	0.0000	0.0006	0.0637	0.0009	0.0003	0.0090	0.0028	0.0017	0.0006	0.1535
			0.0211	0.0070	0.0379	0.0182	0.0002	0.0156	0.0489	0.0016	0.0006	0.0007	0.0020	0.0002	0.0013	0.1283	0.0025	0.0005	0.0188	0.0048	0.0035	0.0014	0.3151

Supplementary Table 9. Free amino acid profiles of ConAgra derived hybrids and B73 for reference. Three replicates of raw flour and air-popped flakes were submitted for analysis (g/100g).

Free Lysine Averages in Ground Flour and Air-Popped Flakes

	**Ground Flour**	***sd***	**Air-Popped Flakes**	***sd***
**H1**	0.0066[bcd]	*0.0029*	0.0041[bc]	*0.0008*
**H2**	0.0026[cd]	*0.0009*	0.0042[bc]	*0.0002*
**H3**	0.0057[bcd]	*0.0018*	0.0042[bc]	*0.0004*
**H4**	0.0090[b]	*0.0037*	0.0032[bc]	*0.0003*
**H5**	0.0063[bcd]	*0.0019*	0.0056[ab]	*0.0008*
average	**0.0060[ab]**	***0.0023***	**0.0042[a]**	***0.0009***
**H6**	0.0079[bc]	*0.0030*	0.0055[ab]	*0.0018*
**H7**	0.0078[bc]	*0.0016*	0.0089[a]	*0.0035*
**H8**	0.0049[bcd]	*0.0004*	0.0047[bc]	*0.0023*
**H9**	0.0039[bcd]	*0.0027*	0.0046[bc]	*0.0018*
**H10**	0.0163[a]	*0.0037*	0.0071[ab]	*0.0002*
average	**0.0082[a]**	***0.0049***	**0.0061[a]**	***0.0018***
**H11**	0.0012[d]	*0.0000*	0.0010[c]	*0.0001*
**H12**	0.0019[cd]	*0.0008*	0.0008[c]	*0.0001*
**H13**	0.0012[d]	*0.0002*	0.0008[c]	*0.0001*
**H14**	0.0015[d]	*0.0005*	0.0009[c]	*0.0001*
**H15**	0.0013[d]	*0.0004*	0.0012[c]	*0.0002*
average	**0.0014[b]**	***0.0003***	**0.00095[b]**	***0.0002***
**B73**	0.0013[d]	*0.0006*	*NA*	*NA*

Supplementary Table 10. Free lysine averages of all popcorn cultivars and B73 in ground flour and air-popped flake samples (g/100g). Significance between cultivar measurements and by group is indicated by lettered superscripts.

Trait	*Effects*	*Degrees of Freedom*	*P-value*
Yield	Location	2	5.359e-11
	Cultivar	14	< 2.2e-16
	Location*Cultivar	28	0.0003732
	Residual	90	
Protein-Bound Lysine in Raw Flour	Cultivar	14	1.926e-07
	Residual	30	
Expansion Volume	Location	2	< 2.2e-16
	Cultivar	14	< 2.2e-16
	Location*Cultivar	28	0.0001407
	Residual	90	
OCFSI	Location	2	< 2.2e-16
	Cultivar	14	< 2.2e-16
	Location*Cultivar	28	3.81e-05
	Residual	90	
Popability	Location	2	<2e-16
	Cultivar	14	<2e-16
	Location*Cultivar	28	0.0125
	Residual	90	
Vitreousness	Location	2	1.583e-10
	Cultivar	14	< 2.2e-16
	Location*Cultivar	28	0.0001117
	Residual	90	
Flake Morphology	Location	2	0.01458
	Cultivar	14	1.804e-06
	Location*Cultivar	28	0.83820
	Residual	90	

Supplementary Table 11. Analysis of variances for traits utilized in the 2020 Ranking System (and flake morphology).
